# Supplementary material for: The Cargo Receptor NDP52 Initiates Selective Autophagy by Recruiting the ULK Complex to Cytosol-Invading Bacteria
Source: Mol Cell. 2019 Apr 18;74(2):320–329.e6. doi: 10.1016/j.molcel.2019.01.041 (PMC6477152; doi:10.1016/j.molcel.2019.01.041)
Supplement: Document S1. Figures S1–S7 [file mmc1.pdf]

Molecular Cell, Volume *74*

Supplemental Information

The Cargo Receptor NDP52 Initiates Selective Autophagy by Recruiting the ULK Complex to Cytosol-Invading Bacteria

Benjamin J. Ravenhill, Keith B. Boyle, Natalia von Muhlinen, Cara J. Ellison, Glenn R. Masson, Elsje G. Otten, Agnes Foeglein, Roger Williams, and Felix Randow

Figure S1. Related to Figures 1, 2 and 6. Confirmation of siRNA mediated knockdown.

(A, B) HeLa cells transfected with the indicated siRNAs were **(A)** lysed and assayed for protein expression by Western blot using the indicated antibodies or **(B)** lysed, total mRNA extracted and the relative abundance of ULK2 and Actin mRNA assayed by RT-PCR. **(C)** HeLa cells or HEK293ET cells transiently transfected with GFP:OPTN were transfected with the indicated siRNAs, lysed and assayed for protein expression by Western blot using the indicated antibodies.

Figure S2. Related to Figure 2. GST-FIP200 fusion protein expression.

(A) Lysates were harvested from HEK293ET cells transiently transfected with plasmids encoding the indicated Luciferase-tagged proteins and assayed for protein expression by Western blot using anti Renilla Luciferase antibody. **(B)** Bacterial lysates were harvested from bacteria expressing GST:FIP200ΔN1115, GST:NDP52 or GST alone and the GST-fusion protein isolated using glutathione conjugated beads. Following washing the bound protein was eluted and visualised by gel electrophoresis and subsequent Coomassie staining.

Figure S3. Related to Figure 3. Expression of Luciferase and HIS-tagged fusion proteins

(**A-D**) HEK293ET cells transiently transfected with the indicated Luciferase tagged constructs were lysed, Luciferase activity in lysates assessed and were equalised to similar activity per unit volume. Samples were analysed by Western blot using an antibody against *Renilla* luciferase. (**E**) Bacterial lysates were prepared from either untransformed (mock) BL21 *E. coli* or expressing the indicated 6His-fusion proteins, resolved by gel electrophoresis and stained by Coomassie.

Figure S4. Related to Figure 4. Expression of Luciferase fusion proteins

(A,C) HEK293ET cells transiently transfected with the indicated Luciferase tagged constructs were lysed, Luciferase activity in lysates assessed and were equalised to similar activity per unit volume. Samples were analysed by Western blot using an antibody against *Renilla* luciferase. **(B)** Alignment of the SKICH domains of the indicated proteins. Residues conserved between all three domains are in red and those between two of the three in pink. Those residues deviant in both NDP52 and TAX1BP1 from CALCOCO1 selected for functional analysis are indicated with blue stars. **(D)** GST:FIP200ΔN1115 was assessed for its ability to bind either Luciferase:NDP52 WT or A119Q from *E. coli* in the presence or absence of $SINTBAD_{aa5\text{-}85}$, $NAP1_{aa5\text{-}75}$ or mock bacterial lysate by LUMIER assay. Error bars are the mean +/- SD of two independent experiments.

Figure S5. Related to Figure 5. Hydrogen Deuterium Exchange Mass Spectrometry

(A) The indicated FIP200, SINTBAD and NDP52 constructs were purified from bacterial lysates, resolved by gel electrophoresis and stained by Coomassie. Hatched lines indicate where lanes from a single gel were cut and pasted side-by-side. **(B)** Raw data from HDX-MS experiment comparing deuterium incorporation into FIP200ΔN1441 alone or in the presence of $SINTBAD_{aa6\text{-}85}$ and $NDP52_{aa20\text{-}127}$. Individual peptides identified are listed (S, start position; E, end position, z, charge state, #D is the maximum possible deuterium incorporation, retention time (rt), in min), % deuterium incorporation at different times shown (with the standard deviation (SD) of the triplicate repeats), the % difference in HDX calculated and the mass difference as both a percentage and mass are shown. Percentage values greater than 5%, and mass differences greater than 0.5 Da are highlighted blue. **(C)** Deuterium uptake plots of two peptides from HDX-MS binding experiment. Each peptide shows a significant reduction (p=<0.0001) in uptake on formation of the

FIP200/SINTBAD/NDP52 protein complex in comparison to FIP200 alone (Apo). Each point is the mean of three repeats, with error bars relating to standard deviation. Some error bars are smaller than the points.

Figure S6. Related to Figure 6. Complementation with NDP52 alleles and the effect of disruption of FIP200-TBK1 adaptor interactions

(A) HeLa cells stably expressing the indicated GFP-tagged proteins were infected with mCherry-*S.* Typhimurium, fixed at 1 h p.i. and confocal micrographs of representative recruitment of the GFP fusion proteins to bacteria acquired. Scale bar 10 μm. **(B, C)** HeLa cells stably expressing **(B)** FIP200ΔN1115:GFP or **(C)** GFP:SINTBAD either alone or together with the indicated siRNA-resistant alleles of NDP52 were transfected with the indicated siRNAs , lysed and assayed for protein expression by Western blot using the indicated antibodies. **(D)** HeLa cells stably expressing the indicated FIP200:GFP constructs were infected with mCherry-*S.* Typhimurium, fixed at 1 h p.i. and frequency of GFP-positive bacteria were enumerated by eye on a wide-field microscope. Mean ± SD of two independent experiments. **(E)** HeLa cells stably expressing GFP-WIPI1 were transfected with the indicated siRNAs, infected with mCherry-*S.* Typhimurium and frequency of GFP-positive bacteria were enumerated by eye on a wide-field microscope. Mean ± SD of two independent experiments.

Figure S7. Related to Figures 1-6. Initiation of selective autophagy by NDP52.

NDP52 coordinates anti-bacterial autophagy by recruiting the ULK and TBK1 kinase complexes to Galectin-8 on damaged endomembranes and by crosslinking cargo to LC3C-positive phagophore membranes. Depicted are residues, which, as described in this study, are essential for the indicated protein-protein interactions.

Supplementary Figure 1

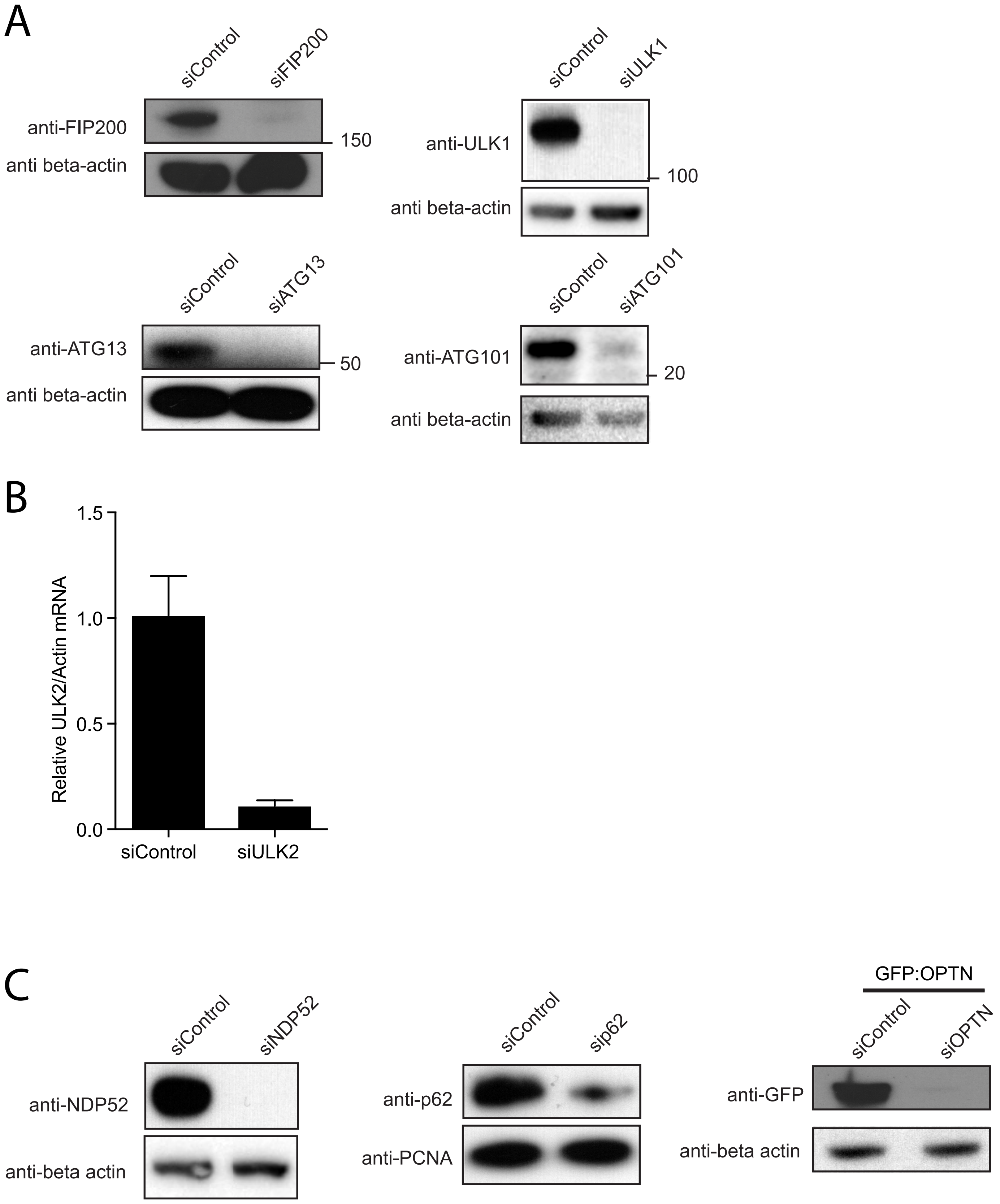

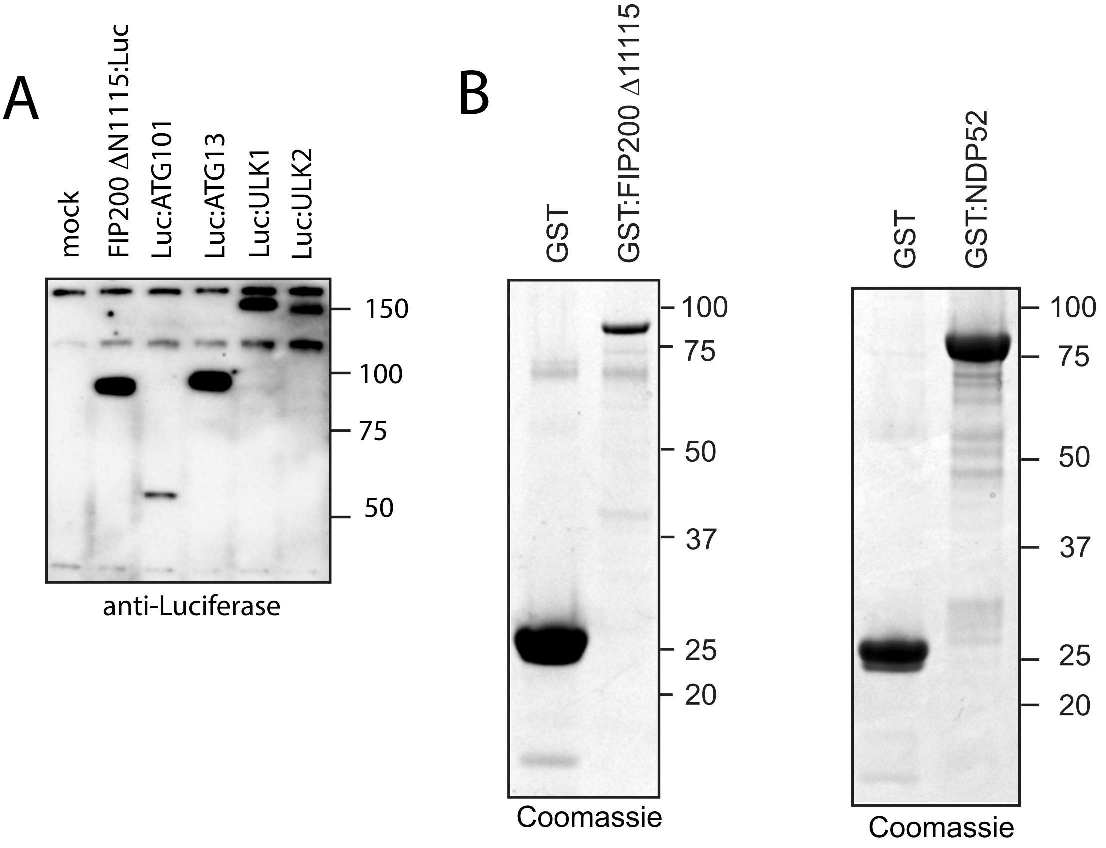
A
mock
FIP200 ΔN1115:Luc
Luc:ATG101
Luc:ATG13
Luc:ULK1
Luc:ULK2
150
100
75
50
anti-Luciferase
B
GST
GST:FIP200 Δ11115
100
75
50
37
25
20
Coomassie
GST
GST:NDP52
100
75
50
37
25
20
Coomassie

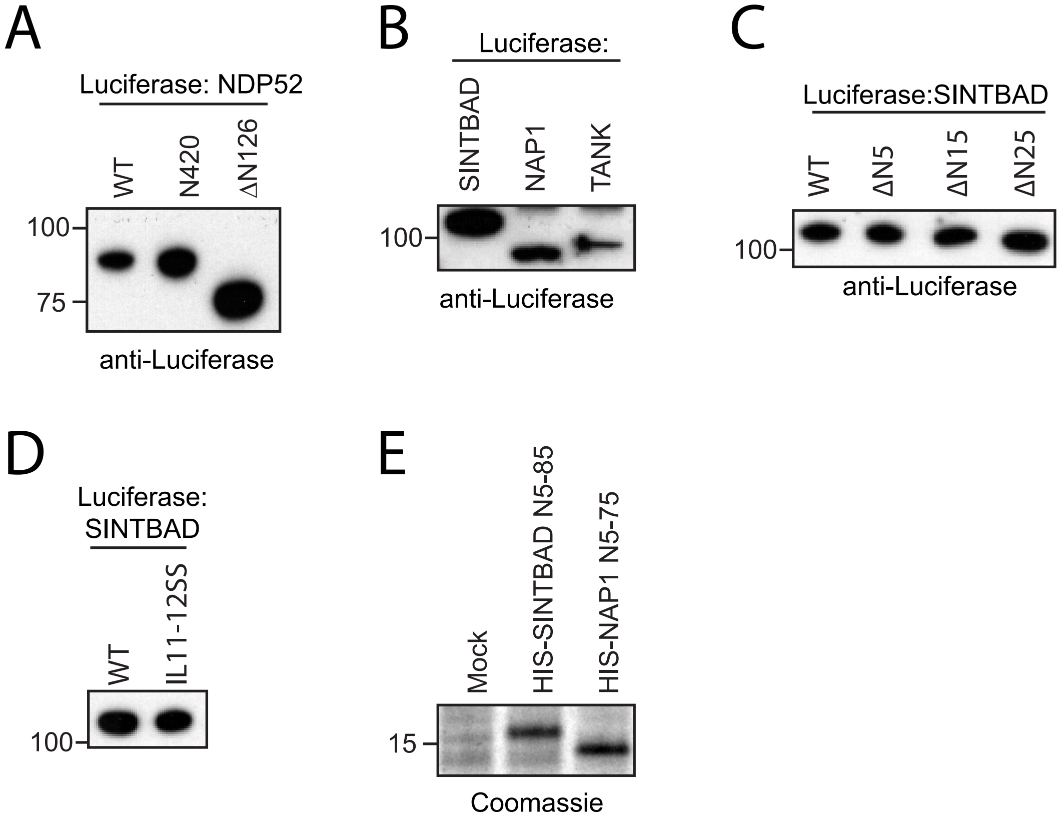
A
Luciferase: NDP52
WT
N420
ΔN126
100
75
anti-Luciferase
B
Luciferase:
SINTBAD
NAP1
TANK
100
anti-Luciferase
C
Luciferase:SINTBAD
WT
ΔN5
ΔN15
ΔN25
100
anti-Luciferase
D
Luciferase:
SINTBAD
WT
IL11-12SS
100
E
Mock
HIS-SINTBAD N5-85
HIS-NAP1 N5-75
15
Coomassie

Supplementary Figure 4

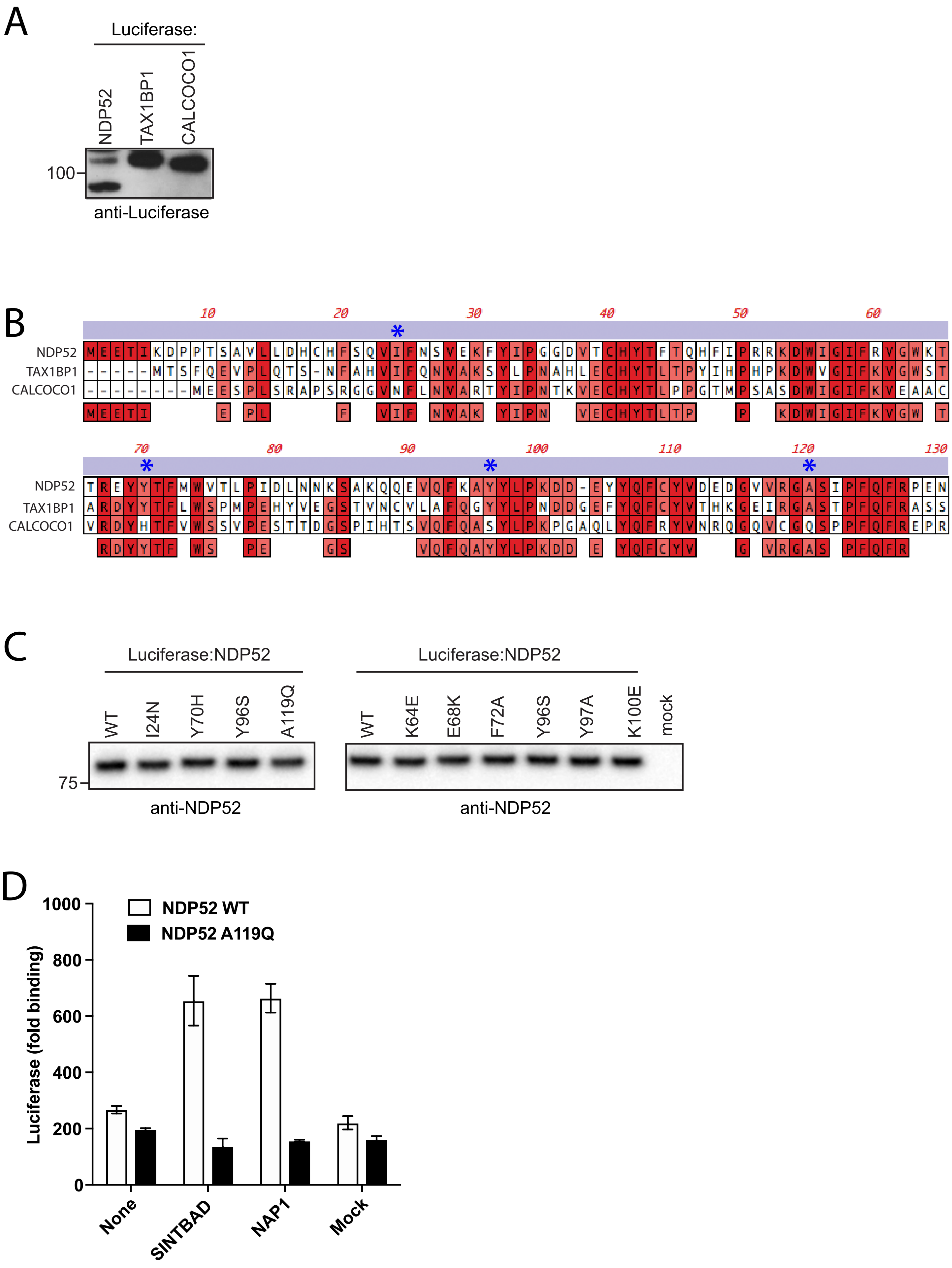

Supplementary Figure 5

A

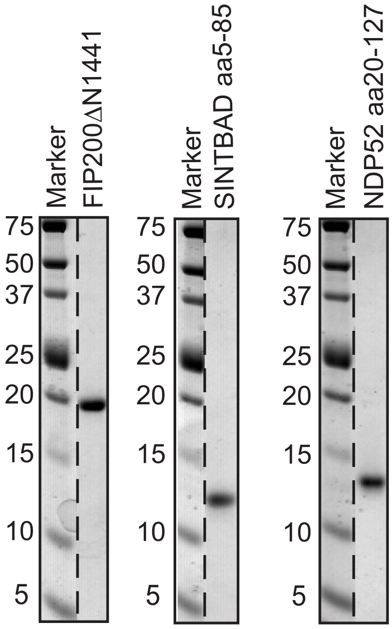

B

Peptide					FIP200						FIP200+SINTBAD +NDP52						% difference in HDX						Da difference in HDX						Sequence
S	E	z	#D	rt (min)	0.3 s	SD	3 s	SD	30 s	SD	0.3 s	SD	3 s	SD	30 s	SD	0.3 s	SD	3 s	SD	30 s	SD	0.3 s	SD	3 s	SD	30 s	SD	
1443	1451	2	7	9.1	81	2	82	1	80	1	81	1	83	2	83	0	0	3	-1	2	-3	1	0.0	0.2	-0.1	0.2	-0.2	0.1	MSVQENIHM
1448	1461	3	12	9.5	31	1	46	1	56	1	29	1	46	2	57	1	2	2	0	3	-1	2	0.3	0.2	0.0	0.4	-0.1	0.3	NIHMLSEEKQRIML
1452	1461	2	8	8.8	28	1	51	1	69	1	25	2	49	1	67	1	4	2	1	2	2	2	0.3	0.2	0.1	0.2	0.1	0.2	LSEEKQRIML
1452	1462	2	9	10.4	23	0	45	1	67	0	21	2	45	1	68	1	2	2	0	2	-1	1	0.2	0.2	0.0	0.2	-0.1	0.1	LSEEKQRIMLL
1453	1461	2	7	8.8	30	1	55	1	75	0	26	2	53	1	72	2	4	2	2	2	2	2	0.3	0.2	0.1	0.1	0.2	0.1	SEEKQRIML
1453	1462	2	8	8.8	28	1	51	1	69	1	25	2	49	1	67	1	4	2	1	2	2	2	0.3	0.2	0.1	0.2	0.1	0.2	SEEKQRIMLL
1454	1461	2	6	8.7	35	1	58	0	76	1	31	2	56	1	77	1	4	3	2	1	0	1	0.2	0.2	0.1	0.1	0.0	0.1	EEKQRIML
1462	1468	1	5	9.7	6	1	36	2	77	1	5	0	30	0	73	1	1	1	6	2	4	3	0.1	0.0	0.3	0.1	0.2	0.1	LERTLQL
1462	1468	2	5	9.7	6	0	36	2	77	2	5	0	31	1	71	2	1	0	5	3	6	3	0.1	0.0	0.2	0.2	0.3	0.2	LERTLQL
1462	1480	3	17	7.2	8	1	41	2	67	1	6	0	35	2	64	1	2	1	6	3	3	2	0.4	0.2	1.0	0.6	0.6	0.3	LERTLQLKEEENKRLNQRL
1462	1480	4	17	7.3	8	0	41	2	67	1	6	1	36	0	64	2	2	1	5	3	3	3	0.4	0.2	0.8	0.4	0.5	0.4	LERTLQLKEEENKRLNQRL
1463	1468	1	4	9.0	6	0	35	2	77	0	5	1	31	1	72	2	1	1	4	3	5	2	0.1	0.0	0.2	0.1	0.2	0.1	ERTLQL
1463	1480	4	16	7.0	9	0	42	2	68	0	6	1	36	1	65	2	2	1	5	2	2	2	0.4	0.1	0.8	0.3	0.4	0.3	ERTLQLKEEENKRLNQRL
1463	1480	5	16	7.1	10	0	41	1	67	1	7	0	36	1	64	2	2	1	4	2	3	2	0.4	0.1	0.7	0.3	0.5	0.4	ERTLQLKEEENKRLNQRL
1468	1479	3	10	4.9	13	1	48	1	64	0	10	1	44	1	63	0	4	2	4	2	1	1	0.4	0.2	0.4	0.2	0.1	0.1	LKEEENKRLNQR
1468	1480	4	11	5.2	12	2	46	2	62	1	9	1	40	1	61	1	4	3	6	3	2	2	0.4	0.3	0.6	0.3	0.2	0.2	LKEEENKRLNQRL
1469	1480	2	10	4.8	13	1	47	2	63	0	10	1	43	1	62	1	4	2	4	2	1	1	0.4	0.2	0.4	0.2	0.1	0.1	KEEENKRLNQRL
1481	1500	3	18	6.1	30	1	36	2	34	1	30	1	36	3	36	1	1	2	-1	5	-2	2	0.1	0.3	-0.1	0.8	-0.3	0.4	MSQSMSSVSSRHSEKIAIRD
1481	1508	4	26	11.4	13	0	17	1	20	1	11	0	17	1	18	0	3	1	1	2	2	2	0.7	0.2	0.1	0.5	0.5	0.5	MSQSMSSVSSRHSEKIAIRDFQVGDLVL
1486	1506	3	19	9.7	13	3	20	0	25	1	12	0	20	1	24	0	1	4	0	1	1	2	0.2	0.7	0.0	0.2	0.2	0.4	SSVSSRHSEKIAIRDFQVGDL
1488	1500	3	11	4.7	22	1	28	1	28	1	20	0	28	2	28	1	2	2	1	3	0	1	0.2	0.2	0.1	0.3	0.0	0.2	VSSRHSEKIAIRD
1489	1500	3	10	4.6	23	1	29	1	29	1	18	1	28	1	28	1	4	2	1	1	1	2	0.4	0.2	0.1	0.1	0.1	0.2	SSRHSEKIAIRD
1506	1510	1	3	13.4	1	0	5	0	18	0	1	0	5	0	17	0	0	0	0	0	0	1	0.0	0.0	0.0	0.0	0.0	0.0	LVLII
1509	1520	2	10	10.2	11	0	17	0	22	0	10	0	15	1	21	1	2	1	2	1	1	1	0.2	0.1	0.2	0.1	0.1	0.1	IILDERHDNYVL
1512	1520	2	7	9.0	12	1	19	0	23	0	10	0	17	1	23	0	2	1	2	1	0	1	0.1	0.1	0.2	0.1	0.0	0.0	DERHDNYVL
1512	1521	2	8	11.4	10	0	14	2	16	1	7	1	12	0	15	1	2	1	2	2	1	1	0.2	0.1	0.1	0.2	0.1	0.1	DERHDNYVLF
1521	1527	1	4	10.3	35	2	71	2	88	1	26	3	66	1	86	1	9	5	5	3	2	2	0.4	0.2	0.2	0.1	0.1	0.1	FTVSPTL
1522	1528	1	4	9.3	31	2	59	2	70	1	24	2	58	1	69	1	7	4	1	3	0	2	0.3	0.2	0.0	0.1	0.0	0.1	TVSPTLY
1528	1535	2	6	10.3	7	1	19	0	36	1	4	1	13	1	30	2	3	1	6	1	5	2	0.2	0.1	0.4	0.1	0.3	0.1	YFLHSESL
1528	1538	2	8	11.8	11	1	22	1	40	0	9	0	15	1	31	1	2	1	7	1	8	1	0.2	0.1	0.6	0.1	0.7	0.1	YFLHSESLPAL
1539	1561	3	19	9.6	32	1	33	1	36	1	29	1	32	3	33	1	3	2	2	3	2	2	0.6	0.3	0.3	0.6	0.5	0.4	DLKPGEGASGASRRPWVLGKVME
1541	1556	3	13	10.2	46	1	49	0	55	1	43	1	47	2	51	1	3	2	2	3	4	2	0.4	0.2	0.3	0.3	0.5	0.2	KPGEGASGASRRPWVL
1547	1556	2	7	10.1	57	1	63	1	72	1	53	1	60	3	69	2	4	2	3	3	3	3	0.3	0.2	0.2	0.2	0.2	0.2	SGASRRPWVL
1547	1563	4	14	9.0	29	2	31	1	37	1	27	2	29	2	32	2	2	3	2	3	5	3	0.3	0.5	0.3	0.5	0.7	0.4	SGASRRPWVLGKVMEKE
1557	1563	1	5	4.0	17	0	19	2	18	0	19	1	19	1	20	1	-2	2	0	3	-2	1	-0.1	0.1	0.0	0.2	-0.1	0.1	GKVMEKE
1557	1582	4	23	8.3	45	1	52	1	55	0	32	1	41	1	47	1	13	1	11	2	8	1	3.0	0.3	2.5	0.4	1.8	0.2	GKVMEKEYCQAKKAQNRFKVPLGTKF
1562	1582	4	18	7.6	47	1	53	1	56	0	32	1	40	1	46	0	15	2	12	2	10	1	2.8	0.3	2.2	0.4	1.8	0.1	KEYCQAKKAQNRFKVPLGTKF
1568	1582	4	12	7.7	42	0	51	0	55	0	28	1	37	1	43	1	14	1	14	1	12	1	1.7	0.1	1.7	0.2	1.5	0.2	KKAQNRFKVPLGTKF
1583	1590	2	6	9.2	18	1	30	0	33	0	14	1	28	0	32	1	4	2	1	1	2	1	0.2	0.1	0.1	0.0	0.1	0.1	YRVKAVSW
1583	1593	3	9	6.7	36	1	45	0	46	0	32	2	42	2	44	2	4	3	2	2	2	2	0.4	0.2	0.2	0.2	0.2	0.2	YRVKAVSWNKK
1583	1594	3	10	6.8	35	1	43	1	44	0	31	1	42	2	42	2	4	2	2	3	2	2	0.4	0.2	0.2	0.3	0.2	0.2	YRVKAVSWNKKV

C

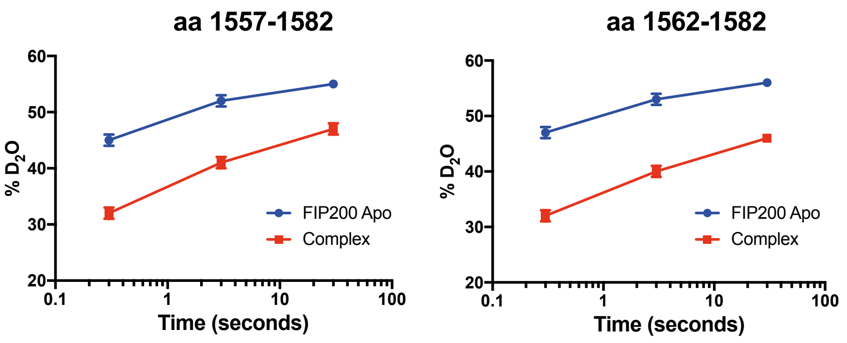

Supplementary Figure 6

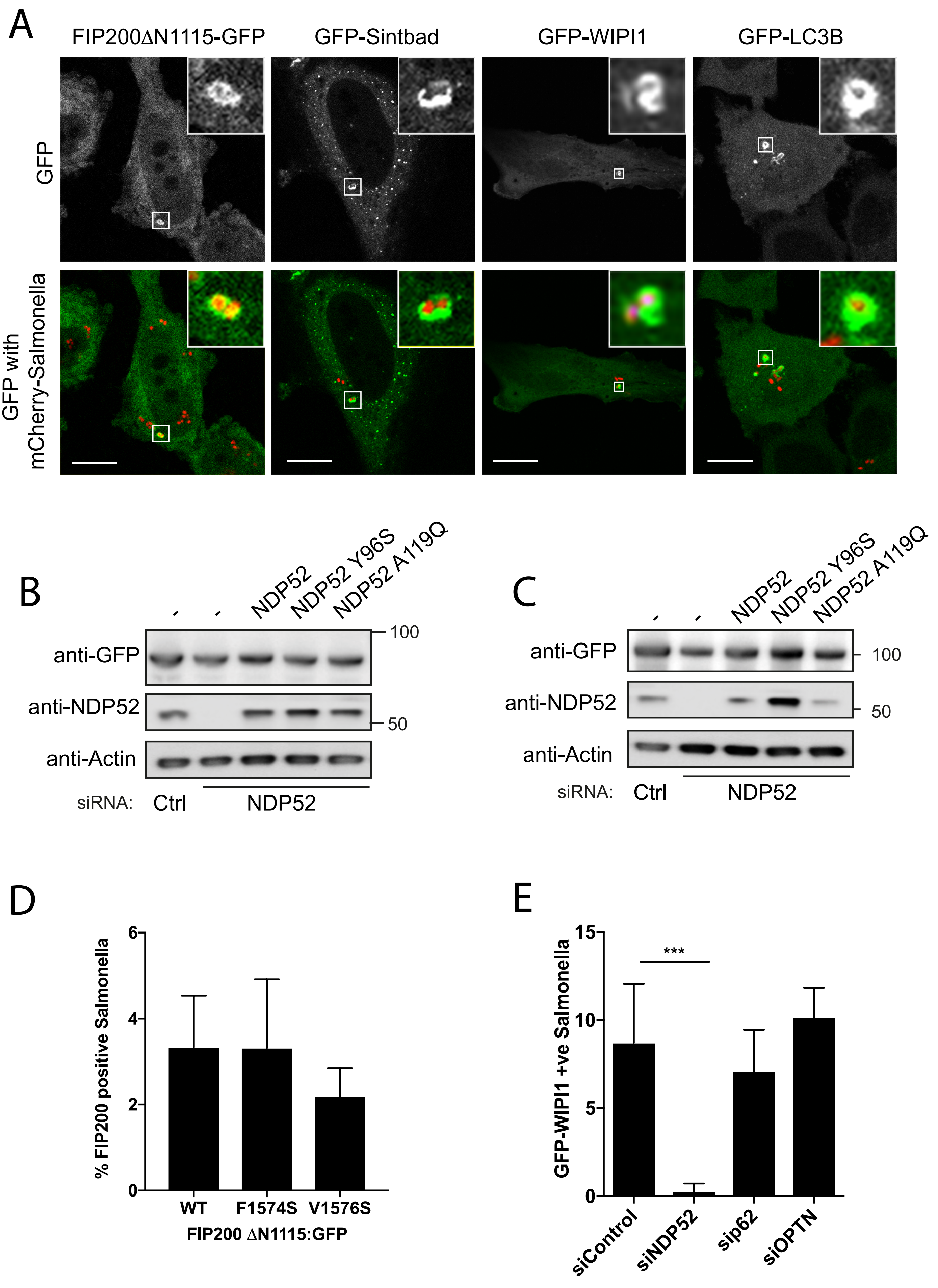

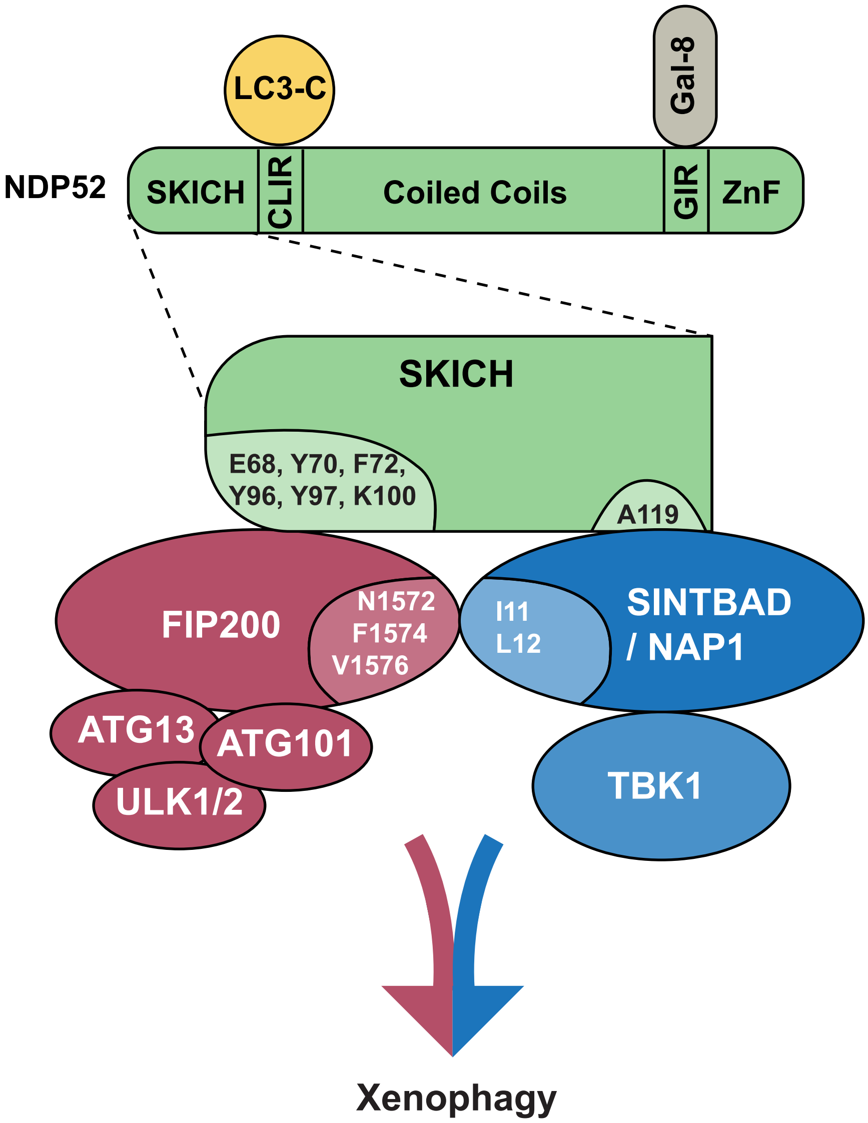
LC3-C
Gal-8
NDP52
SKICH
CLIR
Coiled Coils
GIR
ZnF
SKICH
E68, Y70, F72,
Y96, Y97, K100
A119
FIP200
N1572
F1574
V1576
I11
L12
SINTBAD
/ NAP1
ATG13
ATG101
ULK1/2
TBK1
Xenophagy
