# Supplementary material for: The Cargo Receptor NDP52 Initiates Selective Autophagy by Recruiting the ULK Complex to Cytosol-Invading Bacteria
Source: Mol Cell. 2019 Apr 18;74(2):320–329.e6. doi: 10.1016/j.molcel.2019.01.041 (PMC6477152; doi:10.1016/j.molcel.2019.01.041)
Supplement: Document S2. Article plus Supplemental Information [file mmc2.pdf]

Molecular Cell

Article

The Cargo Receptor NDP52 Initiates Selective Autophagy by Recruiting the ULK Complex to Cytosol-Invading Bacteria

Graphical Abstract

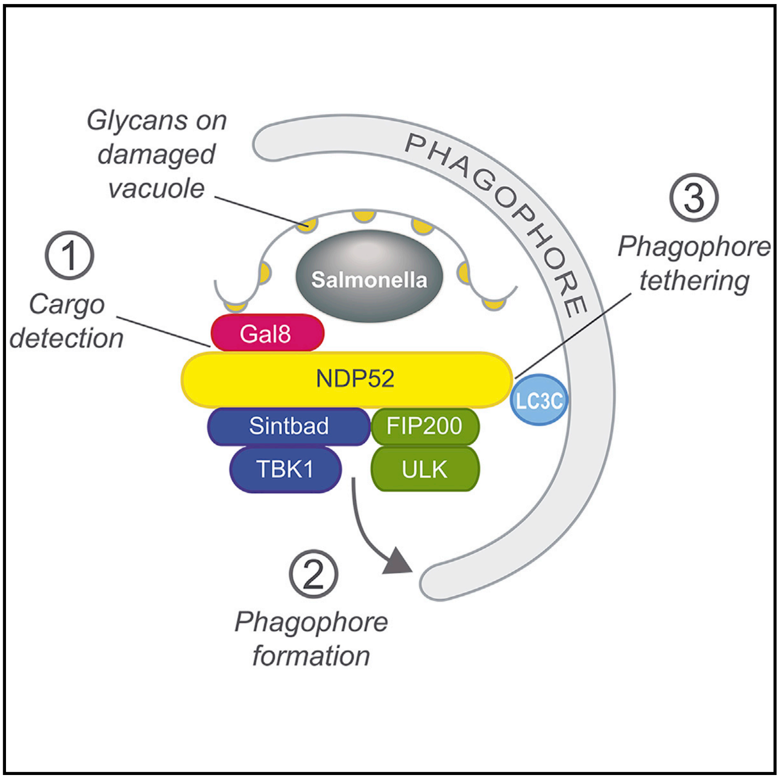

Highlights

- NDP52 recruits upstream autophagy machinery to damaged *Salmonella*-containing vacuoles
- NDP52 trimerizes with the ULK subunit FIP200 and the TBK1 adaptor SINTBAD
- NDP52-dependent recruitment of FIP200-ULK and SINTBAD-TBK1 required for xenophagy
- Recruitment of ULK and TBK1 complexes promotes phagophore formation *in situ*

Authors

Benjamin J. Ravenhill, Keith B. Boyle, Natalia von Muhlinen, ..., Agnes Foeglein, Roger Williams, Felix Randow

Correspondence

randow@mrc-lmb.cam.ac.uk

In Brief

Selective autophagy defends the cytosol against invasive bacteria. In this study, Ravenhill et al. report that the cargo receptor NDP52 recruits the upstream autophagy machinery to cytosolic bacteria by trimerizing with FIP200 and SINTBAD, subunits of the ULK and TBK1 complexes, respectively.

Ravenhill et al., 2019, Molecular Cell *74*, 320–329
April 18, 2019 © 2019 MRC Laboratory of Molecular Biology.
Published by Elsevier Inc.
https://doi.org/10.1016/j.molcel.2019.01.041

CellPress

Molecular Cell
Article

The Cargo Receptor NDP52 Initiates Selective Autophagy by Recruiting the ULK Complex to Cytosol-Invading Bacteria

Benjamin J. Ravenhill,[1,3] Keith B. Boyle,[1,3] Natalia von Muhlinen,[1] Cara J. Ellison,[1] Glenn R. Masson,[1] Elsje G. Otten,[1] Agnes Foeglein,[1] Roger Williams,[1] and Felix Randow[1,2,4,*]
[1]Division of Protein and Nucleic Acid Chemistry, MRC Laboratory of Molecular Biology, Francis Crick Avenue, Cambridge CB2 0QH, UK
[2]Addenbrooke's Hospital, Department of Medicine, University of Cambridge, Cambridge CB2 0QQ, UK
[3]These authors contributed equally
[4]Lead Contact
*Correspondence: randow@mrc-lmb.cam.ac.uk
https://doi.org/10.1016/j.molcel.2019.01.041

SUMMARY

Xenophagy, a selective autophagy pathway that protects the cytosol against bacterial invasion, relies on cargo receptors that juxtapose bacteria and phagophore membranes. Whether phagophores are recruited from a constitutive pool or are generated *de novo* at prospective cargo remains unknown. Phagophore formation *in situ* would require recruitment of the upstream autophagy machinery to prospective cargo. Here, we show that, essential for anti-bacterial autophagy, the cargo receptor NDP52 forms a trimeric complex with FIP200 and SINTBAD/NAP1, which are subunits of the autophagy-initiating ULK and the TBK1 kinase complex, respectively. FIP200 and SINTBAD/NAP1 are each recruited independently to bacteria via NDP52, as revealed by selective point mutations in their respective binding sites, but only in their combined presence does xenophagy proceed. Such recruitment of the upstream autophagy machinery by NDP52 reveals how detection of cargo-associated "eat me" signals, induction of autophagy, and juxtaposition of cargo and phagophores are integrated in higher eukaryotes.

INTRODUCTION

Macroautophagy, a major degradative pathway in eukaryotic cells, is essential for cellular homeostasis. Upon induction of macroautophagy, for example, by starvation, cells deploy a hierarchy of autophagy genes (ATGs) to generate phagophore membranes, which, during their maturation into autophagosomes, entrap cytosol and cytosolic organelles for subsequent delivery to lysosomes (Dikic and Elazar, 2018; Mizushima et al., 2011). In contrast to starvation-induced macroautophagy, which degrades cytosolic components indiscriminately, selective autophagy relies on cargo receptors that detect "eat me" signals associated specifically with cargo destined for degradation (Boyle and Randow, 2013; Stolz et al., 2014). In addition to eat me signals, cargo receptors also bind LC3 and GABARAP proteins, a family of ubiquitin-like proteins associated with phagophores via lipid anchors, which enables the selective uptake of cargo through juxtaposition with phagophore membranes (Slobodkin and Elazar, 2013).

An important function of selective macroautophagy is the protection of the host cytosol from bacterial invasion by, for example, *Salmonella enterica* serovar Typhimurium (*S.* Typhimurium), an enterobacterium that causes more than 100 million infections and 150,000 deaths annually (Benjamin et al., 2013; Deretic et al., 2013; Majowicz et al., 2010; Randow et al., 2013). Upon contact with host cells, *S.* Typhimurium establishes its primary intracellular niche in a membrane-surrounded organelle known as the *Salmonella*-containing vacuole (SCV), either upon phagocytosis or by injecting effector proteins through a type III secretion system into otherwise non-phagocytic cells. To enter the host cytosol, where *S.* Typhimurium proliferates vigorously unless antagonized by xenophagy, bacteria need to cross the limiting SCV membrane, a process that causes extensive membrane damage and thereby exposure of host glycans otherwise hidden inside the SCV (Paz et al., 2010; Thurston et al., 2012). Glycan exposure triggers accumulation of galectin-8 on damaged SCVs, an eat me signal, and ligand for the cargo receptor NDP52 (Thurston et al., 2009, 2012). Subsequent to cytosolic entry, a second type of eat me signal is generated by LUBAC (Noad et al., 2017; van Wijk et al., 2017), LRSAM1 (Huett et al., 2012), PARKIN (Manzanillo et al., 2013), and other host E3 ubiquitin ligases, which coat the bacterial surface with poly-ubiquitin for detection by multiple ubiquitin-binding cargo receptors, including NDP52 (Thurston et al., 2009), optineurin (Wild et al., 2011), and p62 (Zheng et al., 2009).

The current model of selective autophagy emphasizes the importance of cargo receptors, which, by binding eat me signals and LC3/GABARAP family members, achieve selectivity through juxtaposing cargo and phagophores (Svenning and Johansen, 2013). In contrast, the specific contribution of the phagophore-generating upstream ATGs for selective autophagy is less well understood (Mercer et al., 2018). Although essential for all forms of selective autophagy, it remains unclear whether the upstream

 © 2019 MRC Laboratory of Molecular Biology. Published by Elsevier Inc.
This is an open access article under the CC BY license (http://creativecommons.org/licenses/by/4.0/).

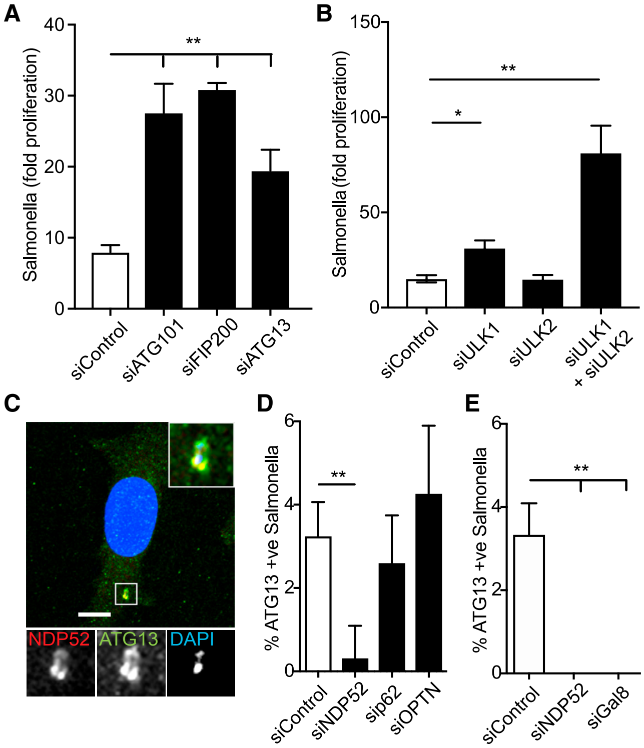

Figure 1. NDP52-Dependent Recruitment of the ULK Complex to *S.* Typhimurium Is Required for Restriction of Bacterial Proliferation

(A) HeLa cells transfected with the indicated siRNAs were infected with *S.* Typhimurium. Colony-forming unit assay was used to assess the proliferation of bacteria over time. Data are depicted as fold proliferation of bacteria at 8 h versus 2 h post infection (p.i.). Mean ± SEM of at least three independent experiments is shown.

(B) HeLa cells transfected with the indicated siRNAs were infected with *S.* Typhimurium. Colony-forming unit assay was used to assess the proliferation of bacteria over time. Data are depicted as fold proliferation of bacteria at 8 h versus 2 h post infection (p.i.). Mean ± SEM of at least three independent experiments is shown.

(C–E) HeLa cells infected with *S.* Typhimurium were fixed at 1 h p.i. and stained for endogenous NDP52 and ATG13 (C) or ATG13 alone (D and E).

(C) A representative confocal micrograph is depicted. DAPI signal in inset represents bacteria.

(D) HeLa cells transfected with the indicated siRNAs were infected with *S.* Typhimurium. The frequency of ATG13-positive bacteria in cells transfected with the indicated siRNAs was enumerated on a wide-field microscope by eye.

(E) The frequency of ATG13-positive bacteria in cells transfected with the indicated siRNAs was enumerated on a wide-field microscope by eye.

Mean ± SD from two independent experiments. $^{*}p < 0.05$; $^{**}p < 0.01$; one-way ANOVA with Dunnett's multiple comparisons test. Scale bar, 10 μm.

autophagy machinery produces phagophores on demand near the prospective cargo or whether cargo receptors recruit phagophores from a constitutive pool. Consistent with phagophore formation occurring in the vicinity of the prospective cargo is the occurrence near *S.* Typhimurium of certain upstream autophagy components, such as FIP200, a subunit of the autophagy-initiating ULK complex, the PI3P-binding proteins WIPI1 and WIPI2, and the E3-like ATG5/12/16 complex (Dooley et al., 2014; Kageyama et al., 2011; Thurston et al., 2016). Precisely how bacteria recruit the upstream autophagy machinery remains unknown, although cargo receptors have been implicated in recruitment of phospho-ULK1 to damaged mitochondria (Lazarou et al., 2015). However, the substantial redundancy that exists among cargo receptors during mitophagy precluded the straightforward ascription of function to any given cargo receptor.

Here, we show that the cargo receptor NDP52 forms a trimeric complex with FIP200 and SINTBAD/NAP1, subunits of the autophagy-initiating ULK and the TBK1 kinase complex, respectively, which explains how galectin-8-positive membrane fragments, via NDP52, recruit the upstream autophagy machinery to *S.* Typhimurium. NDP52 alleles that bind only FIP200 or SINTBAD/NAP1 do not promote progression of anti-bacterial autophagy, as demonstrated by lack of WIPI and LC3 recruitment to bacteria, revealing that recruitment of the upstream autophagy machinery to its prospective cargo by NDP52 is essential for anti-bacterial autophagy driven by galectin-8. Selective autophagy is therefore coordinated by receptor and adaptor functions of NDP52, which detects eat me signals and recruits the autophagy-initiating ULK and TBK1 kinase complexes to foster phagophore formation in close proximity to cargo before, ultimately, crosslinking phagophores and cargo.

RESULTS

The Autophagy-Initiating ULK Complex Is Essential for Anti-bacterial Autophagy

Macroautophagy restricts the proliferation of cytosol-invading *S.* Typhimurium, but information remains sparse on the specific function of upstream core ATGs in this process. Because FIP200, a vertebrate-specific subunit of the autophagy-initiating ULK kinase complex, is required for anti-bacterial autophagy (Kageyama et al., 2011), we investigated whether other components of the ULK complex are similarly needed to restrict bacterial proliferation. We infected cells depleted of specific subunits of the ULK complex with *S.* Typhimurium and found that those lacking FIP200, ATG101, or ATG13 failed to antagonize bacterial proliferation (Figures 1A and S1A). Although cells depleted of the kinase ULK1 displayed a relatively modest increase in bacterial proliferation, and depletion of ULK2 alone had no effect, combined depletion of both ULK1 and ULK2 resulted in a synergistic hyper-proliferation phenotype (Figures 1B, S1A, and S1B). We conclude that the ULK complex requires all of its structural and regulatory subunits, in addition to at least one kinase subunit, to enable its anti-bacterial function.

Having established an essential function for the ULK complex in anti-bacterial autophagy, we asked whether the ULK complex is recruited to cytosol-invading bacteria or whether it can execute its essential role in host defense in locations distant from the invader. We therefore monitored the localization of the ULK complex subunit ATG13 upon infection using an antibody against the endogenous protein. We observed recruitment of ATG13 to NDP52-positive, i.e., cytosol-exposed, *S.* Typhimurium (Figures 1C and 1D) and found that such recruitment specifically required both the autophagy cargo receptor NDP52 (Figures 1D and S1C) and its cognate binding protein galectin-8 (Figure 1E). The recruitment of ATG13 to cytosol-invading

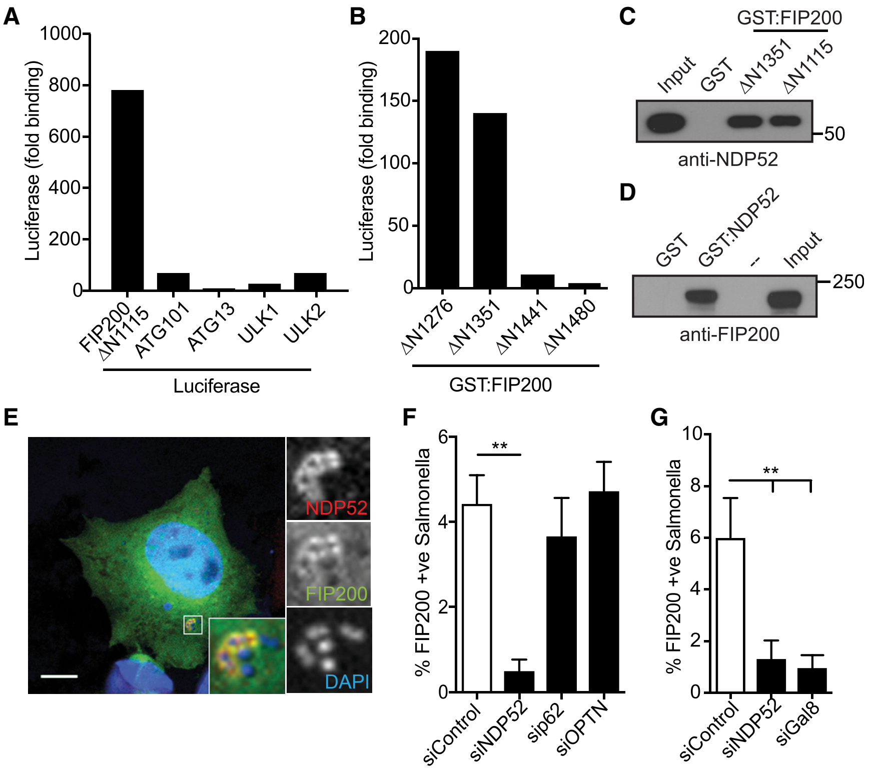

Figure 2. NDP52 Binds FIP200 and Recruits It to Cytosolic *S.* Typhimurium

(A) The indicated Luciferase-tagged constructs were expressed in HEK293ET cells and assessed for their ability to bind glutathione S-transferase (GST):NDP52 purified from *E. coli* in a LUMIER assay.

(B) The indicated C-terminal fragments of GST:FIP200 were expressed in *E. coli* and assessed for their ability to bind Luciferase:NDP52 from HEK293ET cell lysate by LUMIER assay.

(C and D) Bacterially expressed (C) FIP200 fragments or (D) NDP52 were assessed for their ability to bind endogenous NDP52 or FIP200, respectively, from HeLa cell lysates. Input = 10%.

(E–G) Either (E) HeLa cells expressing FIP200ΔN1115:GFP alone or (F and G) transfected with the indicated siRNAs were infected with S. Typhimurium, fixed at 1 h p.i., and stained for endogenous NDP52.

(E) A representative confocal micrograph is depicted.

(F) The frequency of FIP200-positive bacteria was enumerated on a wide-field microscope by eye.

Data in (A) and (B) are expressed as fold binding relative to GST alone. Data are from a single experiment representative of at least two (A–D) or mean ± SD of 2 independent experiments (F and G). $^{**}p < 0.01$; one-way ANOVA with Dunnett's multiple comparisons test. Scale bar, 10 μm.

bacteria by NDP52 suggests that this cargo receptor not only enforces proximity between phagophores and cargo, a function ubiquitously performed by all cargo receptors, but in addition may control upstream steps in selective autophagy, possibly even the induction of phagophore formation.

NDP52 Binds FIP200

To investigate potential upstream roles of NDP52 in selective autophagy, we searched for novel NDP52 interactors by yeast two-hybrid technology. Among 42 clones analyzed, we identified SINTBAD (n = 4) and NDP52 itself (n = 6), two proteins known to bind NDP52 (Thurston et al., 2009), as well as the novel interactor FIP200 (n = 11). We noticed that all FIP200 clones from the yeast two-hybrid screen encoded N-terminally truncated FIP200, suggesting NDP52 binds to the C terminus of FIP200. We confirmed that the yeast two-hybrid fragment FIP200ΔN1115 bound NDP52 using a LUMIER binding assay, and other subunits of the ULK complex did not interact with NDP52 (Figures 2A and S2A). Serial truncation of FIP200 revealed FIP200ΔN1351 as the shortest FIP200 fragment still able to bind NDP52; further truncation of FIP200 (FIP200ΔN1441) abrogated the interaction (Figure 2B). Binding of FIP200ΔN1351 and ΔN1115 to NDP52 was confirmed in cell lysates (Figure 2C), as was the interaction of full-length FIP200 with NDP52 (Figures 2D and S2B).

NDP52 Recruits FIP200 to Cytosol-Invading *S.* Typhimurium

When investigating the distribution of FIP200 in cells infected with *S.* Typhimurium, we found that FIP200 colocalized with NDP52-positive bacteria (Figure 2E). Similar to ATG13, recruitment of FIP200 to bacteria did not require p62 nor Optineurin but was reliant upon NDP52 (Figure 2F) as well as galectin-8 (Figure 2G). Our data reveal that FIP200 is recruited into bacterial proximity by galectin-8 and NDP52, i.e., as a direct consequence of damage to the limiting membrane of bacteria-containing vacuoles rather than due to ubiquitin deposition around bacteria. The binding of FIP200 to and its recruitment by NDP52 to cytosol-invading bacteria suggest an important new function for NDP52 in selective autophagy, namely that it induces antibacterial autophagy *in situ* by recruiting the autophagy-initiating ULK complex for phagophore formation at prospective autophagy cargo.

A Trimeric Complex of FIP200, NDP52, and NAP1/SINTBAD

NDP52 comprises a long central coiled coil domain, flanked by an N-terminal SKICH domain and a C-terminal zinc finger (Figure 3A). We localized the FIP200 binding site in NDP52 by deleting the flanking domains and found that NDP52 requires its SKICH domain, but not its zinc finger, to interact with FIP200 (Figures 3B and S3A).

In addition to FIP200, the SKICH domain of NDP52 also interacts with SINTBAD and NAP1, two closely related TBK1 adaptors (Bouwmeester et al., 2004; Ryzhakov and Randow, 2007; Thurston et al., 2009), suggesting the potential for direct physical interaction between the TBK1 adaptors and FIP200. We therefore tested in a LUMIER binding assay whether NAP1 and SINTBAD also engage FIP200. Indeed, SINTBAD and to a lesser degree NAP1, but not the more distantly related TBK1 adaptor TANK, bound the C terminus of FIP200 (Figures 3C and S3B).

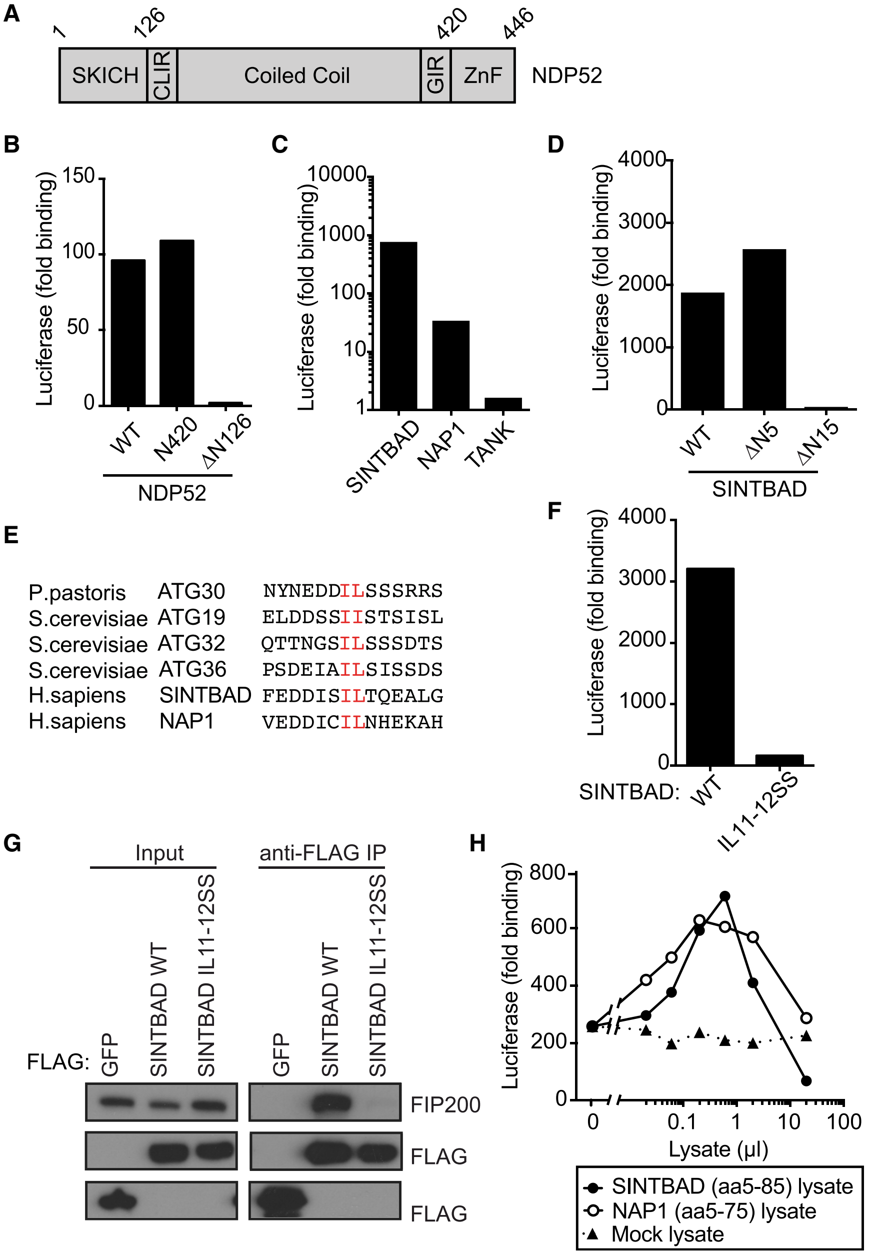

Figure 3. FIP200 Binds the TBK1 Adaptor Proteins SINTBAD and NAP1

(A) Domain structure of NDP52. CLIR, LC3C-specific LC3-interacting region; GIR, galectin-8-interacting region; ZnF, zinc fingers.

(B) Bacterially expressed GST-FIP200ΔN1115 was assessed for binding to the indicated Luciferase-tagged proteins from HEK293ET cell lysates by LUMIER assay. Data are from a single experiment representative of at least two independent experiments.

(C) Bacterially expressed GST-FIP200ΔN1115 was assessed for binding to the indicated Luciferase-tagged proteins from HEK293ET cell lysates by LUMIER assay. Data are from a single experiment representative of at least two independent experiments.

(D) Bacterially expressed GST-FIP200ΔN1115 was assessed for binding to the indicated Luciferase-tagged proteins from HEK293ET cell lysates by LUMIER assay. Data are from a single experiment representative of at least two independent experiments.

(E) Alignment of the di-aliphatic amino-acid-containing regions of the indicated yeast cargo receptors and human SINTBAD and NAP1. Conserved residues within the motif are in red.

(F) Bacterially expressed GST-FIP200ΔN1115 was assessed for binding to the indicated Luciferase-tagged proteins from HEK293ET cell lysates by LUMIER assay. Data are from a single experiment representative of at least two independent experiments.

(G) The indicated FLAG-tagged constructs were expressed in HeLa cells, immunoprecipitated, and assessed for their ability to bind endogenous FIP200. Data are from a single experiment representative of three.

(H) GST-FIP200ΔN1115 was assessed for its ability to bind Luciferase:NDP52 in the presence or absence of the indicated amounts of bacterial lysates containing SINTBADaa5-85, NAP1aa5-75, or mock by LUMIER assay. Data are expressed as fold binding relative to GST alone and are from a single experiment representative of three.

Further analysis revealed that binding to FIP200 was abrogated upon deletion of the N-terminal fifteen, but not the N-terminal five, amino acids in SINTBAD (Figures 3D and S3C). Residues 5–15 in SINTBAD are therefore essential for the interaction with FIP200. Although FIP200 is not encoded in fungal genomes, its C-terminal domain is homologous to ATG11 (Ohashi and Munro, 2010), a protein essential for selective autophagy in yeast. ATG11 binds a di-aliphatic motif in ATG30, ATG36, and ATG19, the cargo receptors for mitophagy, pexophagy, and the autophagy-related cytosol to vacuole targeting (CVT) pathway, respectively (Farré et al., 2013). We noticed a similar di-aliphatic motif in the N terminus of NAP1 and SINTBAD (Figure 3E) and found that the motif is essential for the interaction of SINTBAD with FIP200, when tested against either a C-terminal fragment (Figures 3F and S3D) or the endogenous full-length protein (Figure 3G). We conclude that binding to a di-aliphatic motif is an evolutionarily conserved feature of ATG11/FIP200.

To test whether FIP200 binds NDP52 and NAP1/SINTBAD simultaneously, we performed LUMIER binding experiments with bacterially expressed proteins (Figures 3H, S2, and S3E). Titration of $SINTBAD_{aa5-85}$ or $NAP1_{aa5-75}$ into reactions containing GST:FIP200ΔN1115 and luciferase:NDP52 resulted in bell-shaped binding curves, indicating the formation of trimeric FIP200-NDP52-SINTBAD or FIP200-NDP52-NAP1 complexes, respectively, at low to moderate SINTBAD or NAP1 concentration that were out-titrated when SINTBAD or NAP1

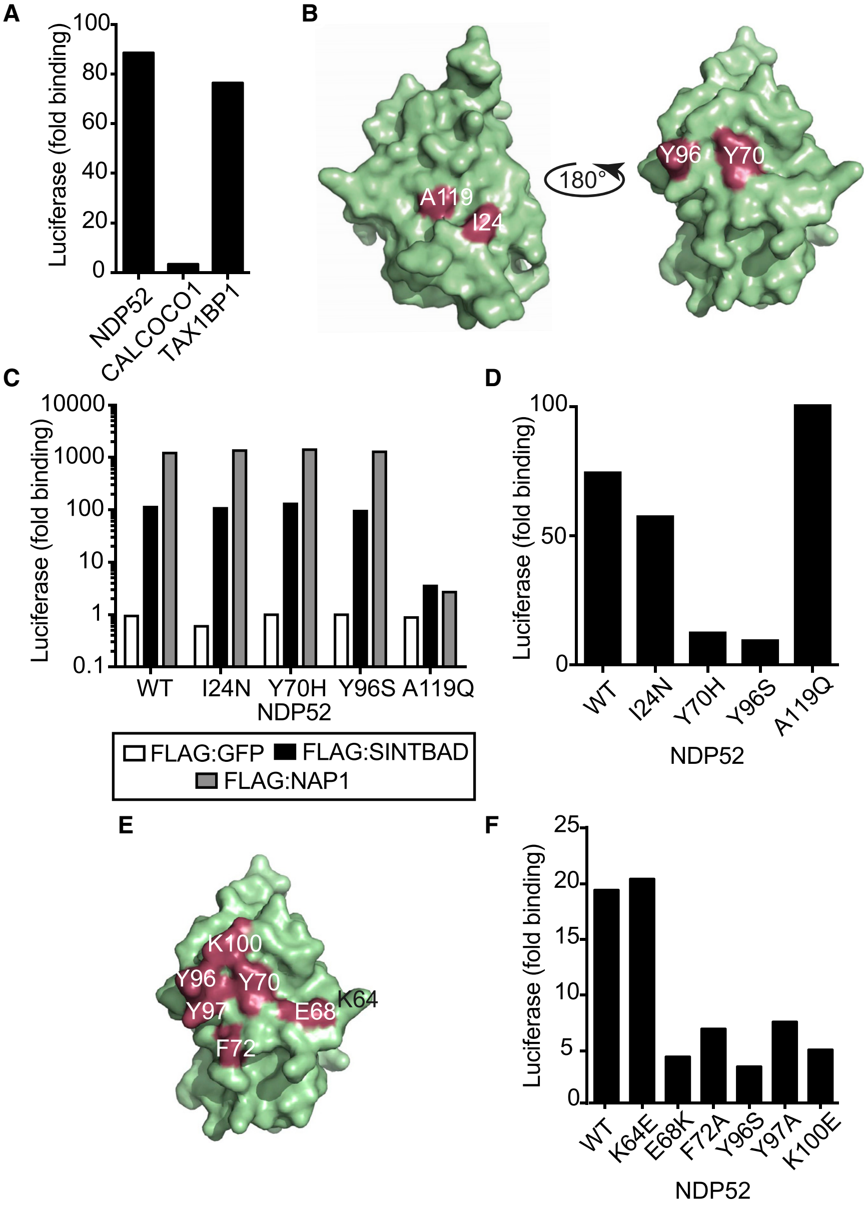

Figure 4. Identification of the Binding Sites for SINTBAD/NAP1 and FIP200 in the NDP52 SKICH Domain

(A) GST:FIP200ΔN1115 was assessed for its ability to bind the indicated Luciferase-tagged proteins from HEK293ET cell lysates by LUMIER assay. Data are expressed as fold binding relative to GST alone and are from a single experiment representative of at least two.

(B) Crystal structure of NDP52 SKICH domain with proposed binding residues highlighted.

(C) The indicated FLAG-tagged proteins were assessed for their ability to bind the indicated Luciferase:NDP52 proteins from HEK293ET cell lysates by LUMIER assay. Data are from a single experiment representative of three independent experiments.

(D) GST:FIP200ΔN1115 was assessed for its ability to bind the indicated Luciferase-tagged proteins from HEK293ET cell lysates by LUMIER assay. Data are expressed as fold binding relative to GST alone and are from a single experiment representative of at least two.

(E) Schematic of NDP52 SKICH domain crystal structure with residues required for binding to FIP200 in red.

(F) GST:FIP200ΔN1115 was assessed for its ability to bind the indicated Luciferase-tagged proteins from HEK293ET cell lysates by LUMIER assay. Data are expressed as fold binding relative to GST alone and are from a single experiment representative of at least two.

concentrations were raised further. These data reveal the existence of a trimeric protein complex, in which the cargo receptor NDP52 binds simultaneously to the ULK complex subunit FIP200 and the TBK1 adaptors SINTBAD/NAP1 and potentially recruits two kinase complexes essential for anti-bacterial autophagy into the vicinity of cytosol-invading bacteria.

Identification of the Binding Surfaces for FIP200 and NAP1/SINTBAD in the NDP52 SKICH Domain

In order to investigate the functional importance of NDP52 in recruiting and juxtaposing FIP200 and NAP1/SINTBAD, we next identified residues essential for the interaction of individual subunits in the trimeric NDP52-FIP200-NAP1/SINTBAD complexes. To identify NDP52$_{SKICH}$ domain residues specifically interacting with FIP200 and NAP1/SINTBAD, we compared the ability of NDP52 and its paralogs TAX1BP1 and CALCOCO1 to bind FIP200. NAP1 and SINTBAD are known to interact with NDP52 and TAX1BP1, but not with CALCOCO1 (Thurston et al., 2009). An identical binding pattern was observed for FIP200 (Figures 4A and S4A). Amino acids conserved in the SKICH domains of NDP52 and TAX1BP1 but deviant in the non-binding CALCOCO1 are therefore candidate residues for controlling SKICH binding to FIP200 and NAP1/SINTBAD (Figure S4B). Mapping candidate residues onto the NDP52$_{SKICH}$ structure (von Muhlinen et al., 2012) revealed two clusters of candidate residues located on opposite faces of the domain (Figure 4B). We next tested the contribution of candidate residues to FIP200 and NAP1/SINTBAD binding by generating NDP52 alleles into which the corresponding CALCOCO1 residues had been introduced. We found that NDP52$_{A119Q}$ failed to bind NAP1 or SINTBAD, although NDP52$_{I24N}$, NDP52$_{Y70H}$, and NDP52$_{Y96S}$ still interacted (Figures 4C and S4C). NDP52$_{A119Q}$ also did not promote the formation of trimeric FIP200-NDP52-SINTBAD or FIP200-NDP52-NAP1 complexes (Figure S4D). In contrast, binding to FIP200 was maintained in NDP52$_{I24N}$ and NDP52$_{A119Q}$ but strongly reduced in NDP52$_{Y70H}$ and NDP52$_{Y96S}$ (Figure 4D). We noticed that, in close proximity to residues essential for FIP200 binding, several surface-exposed aromatic

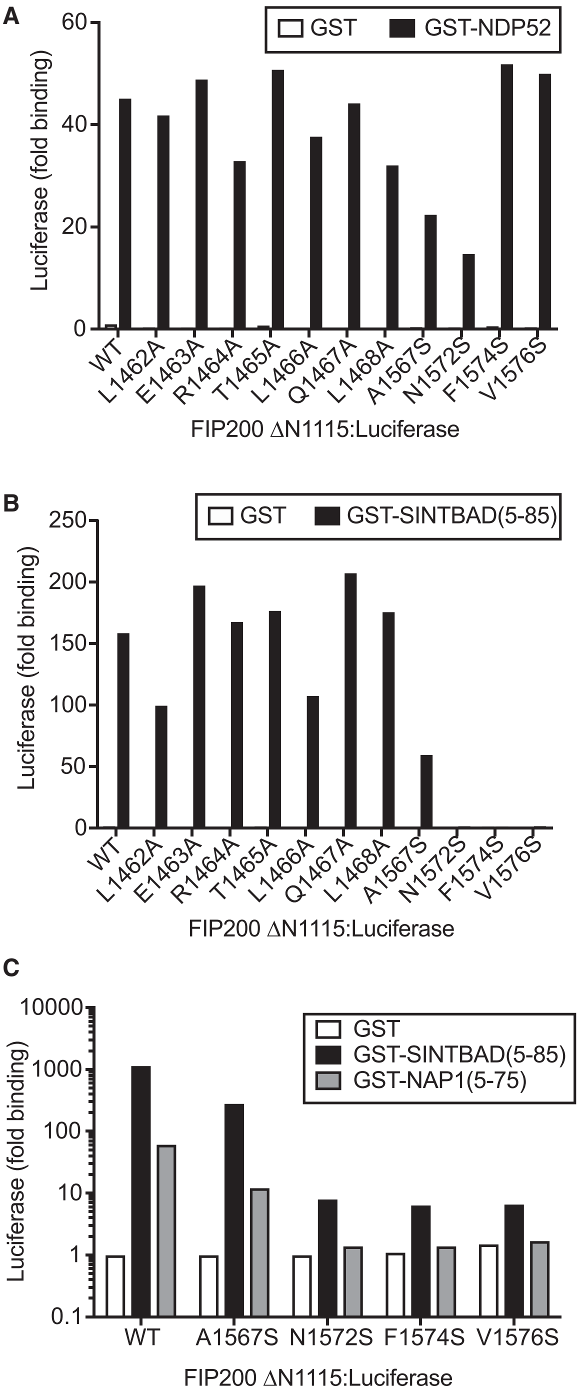

Figure 5. Identification of the Binding Site for SINTBAD/NAP1 in FIP200

(A and B) Bacterially expressed (A) GST:SINTBADaa5-85 or (B) GST:NDP52 were assessed for their ability to bind the indicated FIP200ΔN1115:Luciferase proteins from HEK293ET cell lysates by LUMIER assay.

(C) GST:SINTBADaa5–85 or GST:NAP1aa5–75 was assessed for their ability to bind the indicated FIP200ΔN1115-Luciferase proteins from HEK293ET cell lysates by LUMIER assay. Data are expressed as binding relative to GST only versus FIP200ΔN1115 wild-type (WT):Luciferase and are from a single experiment representative of (A and B) two or (C) three.

residues occur, which also may contribute to the interaction (Figure 4E). Indeed, LUMIER binding assays revealed that $NDP52_{F72A}$, $NDP52_{Y97A}$, as well as $NDP52_{E68K}$ and $NDP52_{K100E}$, but not $NDP52_{K64E}$, failed to bind FIP200 (Figures 4F and S4C).

Taken together, our data show that residues required for the binding of FIP200 and NAP1/SINTBAD are located on opposite faces of the NDP52 SKICH domain, consistent with the formation of trimeric FIP200-NDP52-NAP1/SINTBAD complexes. We conclude that we identified distinct binding sites for FIP200 and NAP1/SINTBAD in the SKICH domain of NDP52 and that we are in the possession of alleles that selectively abrogate binding of NDP52 to either FIP200 or NAP1/SINTBAD.

Identification of the Binding Surface for NAP1/SINTBAD in FIP200

To identify residues in FIP200 essential for the binding of NAP1/SINTBAD and NDP52, we performed hydrogen-deuterium-exchange mass spectrometry (HDX-MS) using purified proteins (Figure S5A). A dataset of 42 peptides, providing 100% peptide coverage of FIP200ΔN1441 with a mean redundancy of 3.5 peptides per amino acid was obtained (mean SD between technical replicates was 0.99%). Upon incubation with $SINTBAD_{aa8–85}$ and $NDP52_{aa20–127}$, a significant reduction in solvent exchange rate for FIP200ΔN1441 occurred between residues 1,557 and 1,582, with peptide 1,568–1,582 exhibiting a 14% ± 1% reduction in exchange rate (1.7 ± 0.1 Da; Figures S5B and S5C). Reductions in exchange rate also occurred in various peptides between residues 1,462 and 1,480. To test whether protection from HDX resulted directly from binding to NDP52 or SINTBAD, we performed LUMIER binding assays with mutant FIP200ΔN1115 alleles. Alleles designed based on the HDX protection of $FIP200_{1,462–1,468}$ did not differ significantly in binding to either NDP52 or SINTBAD (Figures 5A and 5B). However, alleles designed based on the HDX protection of $FIP200_{1,567–1,576}$, namely $FIP200_{N1572S}$, $FIP200_{N1574S}$, and $FIP200_{V1576S}$, selectively failed to interact with SINTBAD (Figure 5B). The same alleles also lost the ability to bind NAP1 (Figure 5C). We conclude that we have identified FIP200 alleles that selectively fail to bind the TBK1 adaptors NAP1 and SINTBAD while maintaining their binding to NDP52.

Anti-bacterial Autophagy Requires NDP52 to Recruit Both FIP200 and NAP1/SINTBAD

The identification of NDP52 alleles specifically impaired in binding to either FIP200 or to NAP1/SINTBAD, as well as of FIP200 alleles specifically lacking the ability to bind NAP1/SINTBAD, enabled us to interfere with individual protein-protein interactions in the FIP200-NDP52-SINTBAD/NAP complex in order to mechanistically dissect the contribution of its components to anti-bacterial autophagy.

Recruitment of FIP200 and SINTBAD to *S.* Typhimurium requires NDP52, as demonstrated by small interfering RNA (siRNA)-mediated depletion of NDP52 from cells and complementation with wild-type NDP52 (Figures 6A, 6B, and S6A–S6C). In contrast, complementation with $NDP52_{Y96S}$ (deficient in binding FIP200) or $NDP52_{A119Q}$ (deficient in binding SINTBAD/NAP1) selectively failed to restore FIP200 or SINTBAD recruitment, respectively. We conclude that binding to NDP52 is

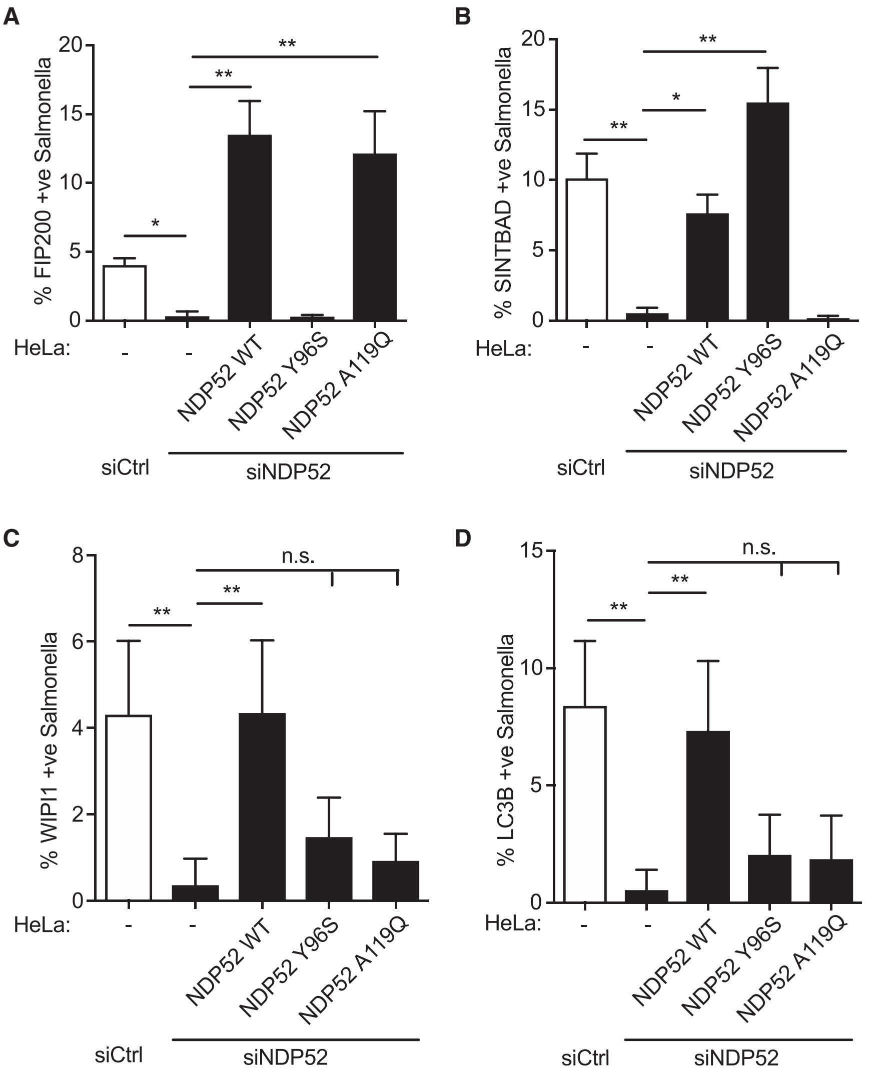

Figure 6. Xenophagy Requires Simultaneous Recruitment of the ULK and SINTBAD/NAP1 Complexes to Cytosolic Bacteria via NDP52

(A–D) HeLa cells stably expressing (A) FIP200ΔN1115:GFP, (B) GFP:SINTBAD, (C) GFP:WIPI1, or (D) GFP:LC3B either alone (−) or together with the indicated NDP52 alleles were transfected with either control or NDP52 siRNA and infected with mCherry-S. Typhimurium for 1 h. Cells were fixed, and the recruitment of GFP-tagged proteins to S. Typhimurium was enumerated on a wide-field microscope by eye. Data are mean ± SEM of three independent experiments (A and B), mean ± SD of 2 independent experiments (C), or representative of at least two independent experiments (D). $^{*}p < 0.05$; $^{**}p < 0.01$; one-way ANOVA with Dunnett's multiple comparisons test.

essential for the recruitment of FIP200 and SINTBAD to cytosol-invading *S.* Typhimurium, and contact between FIP200 and SINTBAD is not required. The non-essential nature of contacts between FIP200 and SINTBAD was confirmed by the unimpaired recruitment to *S.* Typhimurium of FIP200 alleles unable to bind SINTBAD, i.e., $FIP200_{F1574S}$ and $FIP200_{V1576S}$ (Figure S6D).

To test for the functional consequences of NDP52 selectively failing to bind FIP200 or SINTBAD, we investigated the recruitment of components of the downstream autophagy machinery to *S.* Typhimurium in cells complemented with appropriate NDP52 alleles. Recruitment of the phagophore-associated, phosphatidylinositol 3-phosphate (PI3P)-binding protein WIPI1 to *S.* Typhimurium required NDP52, but not p62 or Optineurin (Figures S6A and S6E), a phenotype that was complemented by expression of wild-type NDP52, but not $NDP52_{Y96S}$ or $NDP52_{A119Q}$ (Figure 6C). Cells lacking NDP52 also failed to recruit LC3B, a marker of the phagophore membrane (Figures 6D and S6A). Similar to WIPI1, complementation with wild-type NDP52, but not $NDP52_{Y96S}$ or $NDP52_{A119Q}$, restored LC3B recruitment to *S.* Typhimurium. Taken together, we conclude that FIP200 and SINTBAD/NAP1 are recruited independently by NDP52 to cytosol-invading *S.* Typhimurium but that anti-bacterial autophagy only progresses when NDP52 simultaneously recruits both proteins to *S.* Typhimurium.

Phagophore Formation *In Situ* at Cytosol-Invading *S.* Typhimurium

The requirement for NDP52 to recruit the autophagy-initiating ULK complex to cytosol-invading bacteria suggests that NDP52 initiates anti-bacterial autophagy *in situ*, thus offering an unprecedented opportunity to visualize the early steps of selective autophagy in mammalian cells.

Structural illumination microscopy, a superresolution technique, revealed that galectin-8, a protein that marks the damaged membrane remnants of former *Salmonella*-containing vacuoles, colocalizes tightly with the cargo receptor NDP52 and the ULK complex subunit FIP200 (Figure 7A). Together with biochemical evidence presented earlier, these data suggest that galectin-8 positions NDP52 adjacent to damaged endomembranes, to which it recruits the ULK complex as visualized by FIP200. To investigate the precise localization of the phagophore membrane with respect to the NDP52-FIP200-positive structures, an antibody against endogenous WIPI2 was used. WIPI2 was recruited to the same bacteria that were positive for NDP52 and FIP200. However, the distribution of WIPI2 and NDP52/FIP200 in the bacterial vicinity was distinct (Figure 7B), thus demonstrating the discrete nature of PI3P-positive phagophore membranes and damaged SCVs. Galectin-8, NDP52, and FIP200 usually marked large-membrane patches (Figure 7A), consistent with the nature of broken SCVs, and WIPI2 often formed multiple discontinuous patches around *S.* Typhimurium (Figure 7B). WIPI2 patches frequently appeared as circular structures (yellow arrowheads in Figure 7B), reminiscent of omegasomes,

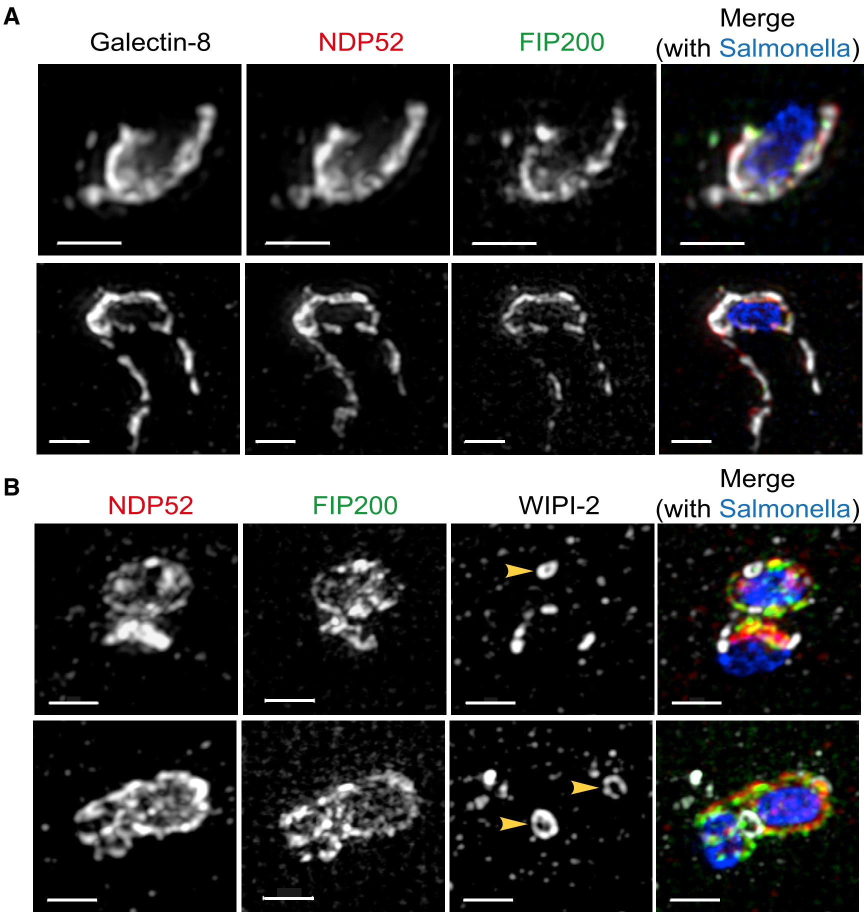

Figure 7. Recruitment of the ULK Complex to Damaged SCV Membranes Initiates Phagophore Formation *In Situ*

(A and B) HeLa cells stably expressing FIP200ΔN1115:GFP were infected with BFP-S. Typhimurium for 1 h, fixed, and stained with (A) anti-galectin-8 and anti-NDP52 antibodies or (B) anti-NDP52 and anti-WIPI2 antibodies. Images were acquired by super-resolution microscopy and are shown as maximum intensity projections. Yellow arrowheads denote WIPI2-positive omegasome-like structures. Scale bars, 1 μm.

indicating that multiple phagophores may form simultaneously adjacent to a single bacterium.

DISCUSSION

The specificity of selective autophagy relies on cargo receptors that juxtapose prospective cargo and phagophores, implying that phagophores are an essential pre-requisite for selective autophagy. However, whether during selective autophagy cargo receptors recruit phagophores from a constitutive pool or deploy phagophores generated *de novo* by the autophagy machinery near the prospective cargo had remained unknown. Here, we show that the recruitment of the upstream autophagy machinery to cytosol-invading bacteria by the cargo receptor NDP52 is essential for phagophore formation and anti-bacterial autophagy (Figure S7). NDP52 forms a trimeric complex with FIP200 and SINTBAD/NAP1, components of the autophagy-initiating ULK complex and the TBK1 kinase complex, respectively, whose combined presence in the bacterial vicinity is required for anti-bacterial autophagy, including the generation of small donut-shaped WIPI structures reminiscent of omegasomes. We conclude that NDP52 provides specificity to selective autophagy at two stages, initially via the recruitment of the upstream autophagy machinery to cargo, resulting in phagophore formation and later via the juxtaposition of phagophores and cargo.

SIM superresolution microscopy revealed tight colocalization of galectin-8, NDP52, and FIP200, indicating their joint recruitment onto membrane remnants of damaged SCVs although, in contrast, WIPI-2 structures that had formed on the same bacterium were topologically distinct and appeared in multiple positions and often as ring-shaped assemblies. The ring-shaped nature of WIPI-2 structures, reminiscent of omegasomes (Roberts and Ktistakis, 2013), suggests that multiple phagophores can form *in situ* on a single bacterium, where they may fuse subsequently to generate a conventional double-membrane autophagosome. The existence of multiple phagophores on a single bacterium and their requirement for membrane fusion may explain how bacteria sometimes become entrapped in onion-like layers comprised of multiple autophagosomal membranes (Zheng et al., 2009).

Cargo receptors are a heterogeneous group of proteins, defined only functionally through their ability to bind members of the LC3/GABARAP family and to sense eat me signals. Individual cargo receptors contain additional domains for potentially unique contributions to selective autophagy, consistent with the identification of FIP200 as a binding partner of NDP52. NDP52 engages FIP200 with its N-terminal SKICH domain, the same domain that also binds the TBK1 adaptors NAP1 and SINTBAD. We have presented biochemical evidence for the formation of a trimeric complex, in which FIP200 and SINTBAD/NAP1 bind to each other as well as to distinct sites in the $NDP52_{SKICH}$ domain. The FIP200 binding patch in $NDP52_{SKICH}$ is formed by several aromatic and charged residues (Y70, F72, Y96, Y97, E68, and K100), which provide a continuous binding surface. The NAP1/SINTBAD binding site is located on the opposite face of the $NDP52_{SKICH}$ domain based on the mutational analysis presented here. Mutants in the two binding sites demonstrate that NDP52 recruits FIP200 and SINTBAD/NAP1 independently to cytosol-invading *S*. Typhimurium and that their combined presence is required for the progression of autophagy. In contrast, interference with binding of FIP200 to SINTBA/NAP1 did not affect recruitment of either protein to *S*. Typhimurium, suggesting

that their direct interaction is not essential for their localization and thus may have other functions. Di-aliphatic motifs similar to the FIP-interacting region (FIR) of NAP1/SINTBAD also occur in ATG19, ATG30, and ATG32, all cargo receptors for selective autophagy in yeast, as well as in CCPG1, a non-canonical cargo receptor essential for selective autophagy of endoplasmic reticulum in vertebrates (Farré et al., 2013; Smith et al., 2018). Identifying the consensus FIR motif, based on further biochemical analysis and structural studies of the known FIP200/ATG11 interactors, may enable the detection of additional proteins with FIR motifs, for example, in novel or known cargo receptors and in other regulators of upstream autophagy.

Although the recruitment of FIP200 via $NDP52_{SKICH}$ is essential for anti-bacterial autophagy, FIR-mediated recruitment of FIP200 may dominate or at least contribute significantly to the uptake of other autophagy cargoes. A similar situation exists for TBK1, whose recruitment in anti-bacterial autophagy is also mediated by $NDP52_{SKICH}$ through the TBK1 adaptors SINTBAD and NAP1, and the direct interaction of TBK1 with the cargo receptor Optineurin may be equally or more important for other cargoes (Morton et al., 2008; Wild et al., 2011). Significant flexibility therefore seems to exist in how cargo receptors recruit the upstream autophagy machinery, thus explaining why, under certain conditions, cargo receptors contribute to autophagy in a redundant fashion. In mitophagy, for example, where cargo receptors are at least partially redundant, mitochondria associated eat me signal(s) are sufficient to recruit multiple cargo receptors, which then engage the upstream autophagy machinery via a network of interactions (Lazarou et al., 2015). In anti-bacterial autophagy, when triggered by glycan exposure and galectin-8 accumulation on damaged endomembranes, NDP52 is essential for autophagy progression and the upstream autophagy machinery is engaged in a unique and non-redundant manner. Subsequently, once the bacterial ubiquitin coat develops, it provides additional eat me signals for the recruitment of other cargo receptors that can engage the autophagy machinery via a network of interactions, similar to mitophagy.

NDP52 is thus a multi-functional cargo receptor, capable of controlling each of the distinct phases of selective autophagy, i.e., cargo recognition (Thurston et al., 2009, 2012), initiation of autophagy (this study), bridging of cargo with the phagophore membrane (von Muhlinen et al., 2012), and, lastly, maturation of the autophagosome (Tumbarello et al., 2012; Verlhac et al., 2015) It remains to be seen whether NDP52 retains further undiscovered functions and whether other cargo receptors are equally multi-functional or perform more specialized roles.

STAR★METHODS

Detailed methods are provided in the online version of this paper and include the following:

- KEY RESOURCES TABLE
- CONTACT FOR REAGENT AND RESOURCE SHARING
- EXPERIMENTAL MODEL AND SUBJECT DETAILS
  - Cell lines
  - Bacteria
- METHOD DETAILS
  - Plasmids
  - Infection with S. Typhimurium and Colony Forming Unit Assay
  - RNA interference
  - Microscopy
  - Immunoprecipitation
  - Western blotting
  - LUMIER assay
  - Yeast Two Hybrid
  - Reverse Transcriptase PCR
  - Protein purification
  - Hydrogen-Deuterium Exchange Mass Spectrometry
- QUANTIFICATION AND STATISTICAL ANALYSIS

SUPPLEMENTAL INFORMATION

Supplemental Information can be found with this article online at https://doi.org/10.1016/j.molcel.2019.01.041.

ACKNOWLEDGMENTS

This work was supported by the MRC (U105170648) and the Wellcome Trust (WT104752MA). B.J.R. was supported by a stipend from the Jean Shanks Foundation.

AUTHOR CONTRIBUTIONS

B.J.R. and K.B.B. planned, performed, and analyzed all experiments with assistance from E.G.O., except the yeast two-hybrid screen (N.v.M.), the structural illumination microscopy (SIM) imaging (C.J.E. and A.F.), and the HDX-MS analysis (G.R.M. and R.W.). F.R. obtained funding and wrote the manuscript with input from all authors.

DECLARATION OF INTERESTS

The authors declare no competing interests.

Received: June 1, 2018
Revised: November 19, 2018
Accepted: January 29, 2019
Published: March 7, 2019

REFERENCES

Benjamin, J.L., Sumpter, R., Jr., Levine, B., and Hooper, L.V. (2013). Intestinal epithelial autophagy is essential for host defense against invasive bacteria. Cell Host Microbe *13*, 723–734.

Bouwmeester, T., Bauch, A., Ruffner, H., Angrand, P.-O., Bergamini, G., Croughton, K., Cruciat, C., Eberhard, D., Gagneur, J., Ghidelli, S., et al. (2004). A physical and functional map of the human TNF-α/NF-κ B signal transduction pathway. Nat. Cell Biol. *6*, 97–105.

Boyle, K.B., and Randow, F. (2013). The role of 'eat-me' signals and autophagy cargo receptors in innate immunity. Curr. Opin. Microbiol. *16*, 339–348.

Deretic, V., Saitoh, T., and Akira, S. (2013). Autophagy in infection, inflammation and immunity. Nat. Rev. Immunol. *13*, 722–737.

Dikic, I., and Elazar, Z. (2018). Mechanism and medical implications of mammalian autophagy. Nat. Rev. Mol. Cell Biol. *19*, 349–364.

Dooley, H.C., Razi, M., Polson, H.E.J., Girardin, S.E., Wilson, M.I., and Tooze, S.A. (2014). WIPI2 links LC3 conjugation with PI3P, autophagosome formation, and pathogen clearance by recruiting Atg12-5-16L1. Mol. Cell *55*, 238–252.

Farré, J.-C., Burkenroad, A., Burnett, S.F., and Subramani, S. (2013). Phosphorylation of mitophagy and pexophagy receptors coordinates their interaction with Atg8 and Atg11. EMBO Rep. *14*, 441–449.

Huett, A., Heath, R.J., Begun, J., Sassi, S.O., Baxt, L.A., Vyas, J.M., Goldberg, M.B., and Xavier, R.J. (2012). The LRR and RING domain protein LRSAM1 is an E3 ligase crucial for ubiquitin-dependent autophagy of intracellular Salmonella Typhimurium. Cell Host Microbe *12*, 778–790.

Kageyama, S., Omori, H., Saitoh, T., Sone, T., Guan, J.-L., Akira, S., Imamoto, F., Noda, T., and Yoshimori, T. (2011). The LC3 recruitment mechanism is separate from Atg9L1-dependent membrane formation in the autophagic response against Salmonella. Mol. Biol. Cell *22*, 2290–2300.

Lazarou, M., Sliter, D.A., Kane, L.A., Sarraf, S.A., Wang, C., Burman, J.L., Sideris, D.P., Fogel, A.I., and Youle, R.J. (2015). The ubiquitin kinase PINK1 recruits autophagy receptors to induce mitophagy. Nature *524*, 309–314.

Majowicz, S.E., Musto, J., Scallan, E., Angulo, F.J., Kirk, M., O'Brien, S.J., Jones, T.F., Fazil, A., and Hoekstra, R.M.; International Collaboration on Enteric Disease 'Burden of Illness' Studies (2010). The global burden of nontyphoidal Salmonella gastroenteritis. Clin. Infect. Dis. *50*, 882–889.

Manzanillo, P.S., Ayres, J.S., Watson, R.O., Collins, A.C., Souza, G., Rae, C.S., Schneider, D.S., Nakamura, K., Shiloh, M.U., and Cox, J.S. (2013). The ubiquitin ligase parkin mediates resistance to intracellular pathogens. Nature *501*, 512–516.

Mercer, T.J., Gubas, A., and Tooze, S.A. (2018). A molecular perspective of mammalian autophagosome biogenesis. J. Biol. Chem. *293*, 5386–5395.

Mizushima, N., Yoshimori, T., and Ohsumi, Y. (2011). The role of Atg proteins in autophagosome formation. Annu. Rev. Cell Dev. Biol. *27*, 107–132.

Morton, S., Hesson, L., Peggie, M., and Cohen, P. (2008). Enhanced binding of TBK1 by an optineurin mutant that causes a familial form of primary open angle glaucoma. FEBS Lett. *582*, 997–1002.

Noad, J., von der Malsburg, A., Pathe, C., Michel, M.A., Komander, D., and Randow, F. (2017). LUBAC-synthesized linear ubiquitin chains restrict cytosol-invading bacteria by activating autophagy and NF-κB. Nat. Microbiol. *2*, 17063.

Ohashi, Y., and Munro, S. (2010). Membrane delivery to the yeast autophagosome from the Golgi-endosomal system. Mol. Biol. Cell *21*, 3998–4008.

Paz, I., Sachse, M., Dupont, N., Mounier, J., Cederfur, C., Enninga, J., Leffler, H., Poirier, F., Prevost, M.-C., Lafont, F., and Sansonetti, P. (2010). Galectin-3, a marker for vacuole lysis by invasive pathogens. Cell. Microbiol. *12*, 530–544.

Randow, F., and Sale, J.E. (2006). Retroviral transduction of DT40. Subcell. Biochem. *40*, 383–386.

Randow, F., MacMicking, J.D., and James, L.C. (2013). Cellular self-defense: how cell-autonomous immunity protects against pathogens. Science *340*, 701–706.

Roberts, R., and Ktistakis, N.T. (2013). Omegasomes: PI3P platforms that manufacture autophagosomes. Essays Biochem. *55*, 17–27.

Ryzhakov, G., and Randow, F. (2007). SINTBAD, a novel component of innate antiviral immunity, shares a TBK1-binding domain with NAP1 and TANK. EMBO J. *26*, 3180–3190.

Slobodkin, M.R., and Elazar, Z. (2013). The Atg8 family: multifunctional ubiquitin-like key regulators of autophagy. Essays Biochem. *55*, 51–64.

Smith, M.D., Harley, M.E., Kemp, A.J., Wills, J., Lee, M., Arends, M., von Kriegsheim, A., Behrends, C., and Wilkinson, S. (2018). CCPG1 is a non-canonical autophagy cargo receptor essential for ER-phagy and pancreatic ER proteostasis. Dev. Cell *44*, 217–232.e11.

Stolz, A., Ernst, A., and Dikic, I. (2014). Cargo recognition and trafficking in selective autophagy. Nat. Cell Biol. *16*, 495–501.

Svenning, S., and Johansen, T. (2013). Selective autophagy. Essays Biochem. *55*, 79–92.

Thurston, T.L., Ryzhakov, G., Bloor, S., von Muhlinen, N., and Randow, F. (2009). The TBK1 adaptor and autophagy receptor NDP52 restricts the proliferation of ubiquitin-coated bacteria. Nat. Immunol. *10*, 1215–1221.

Thurston, T.L., Wandel, M.P., von Muhlinen, N., Foeglein, A., and Randow, F. (2012). Galectin 8 targets damaged vesicles for autophagy to defend cells against bacterial invasion. Nature *482*, 414–418.

Thurston, T.L., Boyle, K.B., Allen, M., Ravenhill, B.J., Karpiyevich, M., Bloor, S., Kaul, A., Noad, J., Foeglein, A., Matthews, S.A., et al. (2016). Recruitment of TBK1 to cytosol-invading Salmonella induces WIPI2-dependent antibacterial autophagy. EMBO J. *35*, 1779–1792.

Tumbarello, D.A., Waxse, B.J., Arden, S.D., Bright, N.A., Kendrick-Jones, J., and Buss, F. (2012). Autophagy receptors link myosin VI to autophagosomes to mediate Tom1-dependent autophagosome maturation and fusion with the lysosome. Nat. Cell Biol. *14*, 1024–1035.

van Wijk, S.J.L., Fricke, F., Herhaus, L., Gupta, J., Hötte, K., Pampaloni, F., Grumati, P., Kaulich, M., Sou, Y.-S., Komatsu, M., et al. (2017). Linear ubiquitination of cytosolic Salmonella Typhimurium activates NF-κB and restricts bacterial proliferation. Nat. Microbiol. *2*, 17066.

Verlhac, P., Grégoire, I.P., Azocar, O., Petkova, D.S., Baguet, J., Viret, C., and Faure, M. (2015). Autophagy receptor NDP52 regulates pathogen-containing autophagosome maturation. Cell Host Microbe *17*, 515–525.

von Muhlinen, N., Akutsu, M., Ravenhill, B.J., Foeglein, Á., Bloor, S., Rutherford, T.J., Freund, S.M., Komander, D., and Randow, F. (2012). LC3C, bound selectively by a noncanonical LIR motif in NDP52, is required for antibacterial autophagy. Mol. Cell *48*, 329–342.

Wild, P., Farhan, H., McEwan, D.G., Wagner, S., Rogov, V.V., Brady, N.R., Richter, B., Korac, J., Waidmann, O., Choudhary, C., et al. (2011). Phosphorylation of the autophagy receptor optineurin restricts Salmonella growth. Science *333*, 228–233.

Zheng, Y.T., Shahnazari, S., Brech, A., Lamark, T., Johansen, T., and Brumell, J.H. (2009). The adaptor protein p62/SQSTM1 targets invading bacteria to the autophagy pathway. J. Immunol. *183*, 5909–5916.

STAR★METHODS

KEY RESOURCES TABLE

REAGENT or RESOURCE	SOURCE	IDENTIFIER
Antibodies		
Rabbit polyclonal anti-NDP52	Abcam	Cat# ab68588; RRID:AB_1640255
Rabbit polyclonal anti-NDP52	Gift from John Kendrick-Jones (LMB, Cambridge)	N/A
Mouse polyclonal anti-NDP52	Abnova	Cat# H00010241-B01P; RRID:AB_1571984)
Rabbit polyclonal anti-Beta Actin	Abcam	Cat# ab8227; RRID:AB_2305186
Rabbit monoclonal anti-ATG13 (E1Y9V)	Cell Signaling Tehnology	Cat# 13468
Rabbit polyclonal anti-FIP200	ProteinTech	Cat# 10043-2-AP; RRID:AB_2253571
Mouse monoclonal anti-FLAG M2	Sigma-Aldrich	Cat# F1804; RRID:AB_262044
Goat polyclonal anti-Galectin8	R and D Systems	Cat# AF1305; RRID:AB_2137229
Rabbit polyclonal anti-ATG101	Sigma Aldrich	Cat# SAB4200175; RRID:AB_10640756)
Mouse monoclonal anti-Renilla Luciferase clone 5B11.2	Merck Millipore	Cat# MAB4400; RRID:AB_95116
Mouse monoclonal anti-ULK1	Santa Cruz	Cat# sc-390904
Rabbit polyclonal anti-GFP	Abcam	Cat# ab290;RRID:AB_303395
Mouse anti-p62/SQSTM1	BD Transduction Laboratories	Cat# 610832; RRID:AB_398151
Alexa-conjugated anti-mouse	Thermo Fisher Scientific	N/A
Alexa-conjugated anti-rabbit	Thermo Fisher Scientific	N/A
Alexa-conjugated anti-goat	Thermo Fisher Scientific	N/A
Bacterial and Virus Strains		
Salmonella enterica serovar Typhimurium strain 12023	Gift from David Holden (Imperial College London)	N/A
S. Typhimurium pFPV25.1-mCherry	This study	
S. Typhimurium pFPV25.1-BFP	This study	
Chemicals, Peptides, and Recombinant Proteins		
Lipofectamine RNAiMAX	Thermo Fisher Scientific	
VECTASHIELD HardSet Antifade Mounting Medium with DAPI	Vector Laboratories	Cat# H-1500
ProLong Gold Antifade Mountant	Thermo Fisher Scientific	Cat# P36930
Polyethylenimine (PEI)	Polysciences	Cat# 23966-2
Complete Protease Inhibitor Cocktail	Roche	Cat #4693116001
Gentamicin	Thermo Fisher Scientific	Cat #15750045
Glutathione 4b Sepharose	GE Healthcare Life Sciences	Cat #17-0756-01
Anti-FLAG M2 agarose resin	Sigma Aldrich	Cat #A2220
FLAG peptide	Sigma Aldrich	Cat #F3290
Critical Commercial Assays		
Renilla Luciferase Assay	Promega	Cat #E2810
RNeasy Plus Mini Kit	QIAGEN	Cat# 74134
Amersham ECL	GE Healthcare Life Sciences	Cat# RPN2106
ProQuest Two-Hybrid System		Cat# PQ10001-01
SuperScript III reverse transcriptase kit	Thermo Fisher Scientific	Cat# 18080093
Power SYBR qPCR green kit	Applied Biosystems	Cat# 4368577
Experimental Models: Cell Lines		
HeLa	American Tissue Culture Collection	RRID:CVCL_0030
HEK293ET	Lab strain	RRID:CVCL_6996

(*Continued on next page*)

Continued		
REAGENT or RESOURCE	SOURCE	IDENTIFIER
Oligonucleotides		
Stealth RNAi siRNA Negative Control, Med GC	Thermo Fisher Scientific	Cat# 12935300
Stealth siRNA NDP52 custom design 5′-GGGAGACAGAGCUGCUUCAACUGAA	Thermo Fisher Scientific	N/A
Stealth siRNA FIP200	Thermo Fisher Scientific	Cat# HSS190643
Stealth siRNA ATG101 custom design 5′- GACUGUGACUUCAUCGACUUCACUU	Thermo Fisher Scientific	N/A
Stealth siRNA ATG13 custom design 5′-CCAUGUGUGUGGAGAUUUCACUUAA	Thermo Fisher Scientific	N/A
Stealth siRNA Galectin-8	Thermo Fisher Scientific	Cat# HSS106038
Silencer Select Negative Control No. 1	Thermo Fisher Scientific	Cat# 4390843
Silencer Select siRNA ULK1	Thermo Fisher Scientific	Cat# s15964
Silencer Select siRNA ULK2	Thermo Fisher Scientific	Cat# s18705
siRNA OPTN custom design 5′-CCACCAGCUGAAAGAAGCCUU	Dharmacon	N/A
siRNA p62/SQSTM1	Dharmacon	Cat# D-010230-02-0005
Recombinant DNA		
pETM30-FIP200 ΔN1115	This study	N/A
pETM30-FIP200 ΔN1276	This study	N/A
pETM30-FIP200 ΔN1351	This study	N/A
pETM30-FIP200 ΔN1441	This study	N/A
pETM30-FIP200 ΔN1480	This study	N/A
pETM30-NDP52	This study	N/A
pETM30-NDP52 20-127	This study	N/A
pETM30-SINTBAD 5-85	This study	N/A
pETM11-SINTBAD 5-85	This study	N/A
pETM11 NAP1 5-75	This study	N/A
M5P- FIP200 ΔN1115-Luciferase	This study	N/A
M5P-Luciferase-ATG101	This study	N/A
M5P-Luciferase-ATG13	This study	N/A
M5P-Luciferase-ULK1	This study	N/A
M5P-Luciferase-ULK2	This study	N/A
M5P-Luciferase-NDP52	This study	N/A
M5P-Luciferase-NDP52 N420	This study	N/A
M5P-Luciferase-NDP52 ΔN126	This study	N/A
M5P-Luciferase-SINTBAD ΔN5	This study	N/A
M5P-Luciferase-SINTBAD ΔN15	This study	N/A
M5P-Luciferase-SINTBAD IL11-12SS	This study	N/A
M5P-Luciferase-CALCOCO1	This study	N/A
M5P-Luciferase-TAX1BP1	This study	N/A
M5P-Luciferase-NDP52 I24N	This study	N/A
M5P-Luciferase-NDP52 Y70H	This study	N/A
M5P-Luciferase-NDP52 Y96S	This study	N/A
M5P-Luciferase-NDP52 A119Q	This study	N/A
M5P-Luciferase-NDP52 K64E	This study	N/A
M5P-Luciferase-NDP52 E68K	This study	N/A
M5P-Luciferase-NDP52 F72A	This study	N/A
M5P-Luciferase-NDP52 Y97A	This study	N/A

(*Continued on next page*)

Continued		
REAGENT or RESOURCE	SOURCE	IDENTIFIER
M5P-Luciferase-NDP52 K100E	This study	N/A
M5P-FIP200 ΔN1115 L1462A-Luciferase	This study	N/A
M5P-FIP200 ΔN1115 E1463A-Luciferase	This study	N/A
M5P-FIP200 ΔN1115 R1464A-Luciferase	This study	N/A
M5P-FIP200 ΔN1115 T1465A-Luciferase	This study	N/A
M5P-FIP200 ΔN1115 L1466A-Luciferase	This study	N/A
M5P-FIP200 ΔN1115 Q1467A-Luciferase	This study	N/A
M5P-FIP200 ΔN1115 L1468A-Luciferase	This study	N/A
M5P-FIP200 ΔN1115 A1567S-Luciferase	This study	N/A
M5P-FIP200 ΔN1115 N1572S-Luciferase	This study	N/A
M5P-FIP200 ΔN1115 F1574S-Luciferase	This study	N/A
M5P-FIP200 ΔN1115 V1576S-Luciferase	This study	N/A
M5P-FLAG-GFP	This study	N/A
M5P-FLAG-SINTBAD	This study	N/A
M5P-FLAG-SINTBAD IL11-12SS	This study	N/A
M5P-FLAG-NAP1	This study	N/A
M6P-NDP52-IRES-PAC	This study	N/A
M6P-NDP52 Y96S-IRES-PAC	This study	N/A
M6P-NDP52 A119Q-IRES-PAC	This study	N/A
M6P-GFP-FIP200 ΔN1115-IRES-Bsr (Blasticidin^R)	This study	N/A
M6P-GFP-SINTBAD-IRES-Bsr	This study	N/A
M6P-GFP-WIPI1-IRES-Bsr	This study	N/A
M6P-GFP-LC3B-IRES-Bsr	This study	N/A
Software and Algorithms		
GraphPad Prism	https://www.graphpad.com/scientific-software/prism/	N/A
Zeiss ZEN	https://www.zeiss.com/microscopy/int/products/microscope-software/zen-lite.html	N/A
aCOLyte3	https://www.synbiosis.com/acolyte-software/	N/A
Proteome Discoverer	https://www.thermofisher.com/order/catalog/product/OPTON-30795	N/A
HD-Examiner Software	http://massspec.com/hdexaminer/	N/A
Other		
HIS-Trap chromatography cartridge	Fisher Scientific	Cat# 11773209
PD-10 desalting column	GE Lifesciences	Cat# 17-0851-01
MonoS cation exchange column	GE Lifesciences	Cat# 17518001
HiLoad 16/600 Superdex 75 column	GE Lifesciences	Cat# 28989333
Resource Q anion exchange column	GE Lifesciences	Cat# 17117701
Poroszyme Immobilized Pepsin cartridge	Applied Biosystems	Cat# 2-3131-00
Acquity 1.7 μm particle 100 mm x 1 mm C18 UPLC Column	Waters	Cat# 186002346
Vivaspin concentrators, various molecular weight cut-offs	Sartorius	N/A

CONTACT FOR REAGENT AND RESOURCE SHARING

Further information and requests for reagents may be directed to the Lead Contact, Felix Randow (randow@mrc-lmb.cam.ac.uk).

EXPERIMENTAL MODEL AND SUBJECT DETAILS

Cell lines

HeLa and HEK293ET cells were grown in Iscove's Modified Dulbecco's Medium supplemented with 10% heat-inactivated (56°C for 30 min) fetal calf serum (FCS) and 30 μg/ml gentamicin at 37 °C in 5% CO_2. Both HeLa and HEK293ET cells are of female origin.

Bacteria

S. Typhimurium (strain 12023), provided by D. Holden (Imperial College London), was grown overnight in Luria broth (LB), with 100 μg/ml ampicillin for those strains harboring fluorescent protein expression plasmids, and sub-cultured (1:33) in fresh LB for 3.5 h before infection. *S.* Typhimurium expressing either the fluorescent protein mCherry or BFP from a pFPV25.1 plasmid were used where indicated.

METHOD DETAILS

Plasmids

M5P or closely related plasmids were used for both transient transfection and for the production of recombinant MLV for the stable expression of proteins in mammalian cells (Randow and Sale, 2006). Open reading frames encoding human NDP52, CALCOCO1, T6BP and LC3B were amplified by PCR from a human brain cDNA library. Plasmids harboring human FIP200, ULK1, ULK2, ATG101, ATG13 and WIPI-1 were kindly provided by S. Tooze (Crick Institute, London, UK). SINTBAD, NAP1 and TANK have been described before (Ryzhakov and Randow, 2007). The ΔN1115 and ΔN1351 N-terminal truncations of FIP200 were subcloned from the Yeast-two-Hybrid hit plasmids. Further truncations of FIP200 (ΔN1276, ΔN1441 and ΔN1480) were amplified by PCR. All further truncations and point mutations were introduced by PCR-mediated mutagenesis. pETM11 (for 6xHis tag) or pETM30 (for 6xHis-GST fusion tag) plasmids were used for bacterial protein expression.

Infection with S. Typhimurium and Colony Forming Unit Assay

HeLa cells, grown in 24-well format, were placed in antibiotic-free medium at least 30 min prior to infection with 20 μL *S.* Typhimurium subculture (diluted 1:5 in antibiotic-free IMDM/10% FCS) for 10 min at 37 °C. Following two washes with warm PBS, cells were cultured in 100 μg/ml gentamycin for 1 h and 20 μg/ml gentamycin thereafter. To enumerate intracellular viable bacteria, cells from triplicate wells were lysed in 1 ml PBS containing 0.1% Triton X-100 at either 2 h or 8 h post-infection. Serial dilutions were plated in duplicate on 5 cm LB agar plates and colonies allowed to develop overnight. The number of colonies per plate, lying within the linear range of the assay, was enumerated using a colony counter apparatus and software (Acolyte, Synbiosis).

RNA interference

2×10^4 HeLa cells per well were seeded in 24-well plates. The following day, cells were transfected with either 66 pmol of Stealth siRNA or 6 pmol of Silencer Select siRNA (Life Technologies) using Lipofectamine RNAiMAX (Life Technologies) in full medium. Medium was replaced after 48 h. Experiments were performed after a total of 72h.

Microscopy

HeLa cells were grown on glass coverslips prior to infection. After infection, cells were washed with PBS and fixed in 4% paraformaldehyde/PBS for 20 min. Cells were washed twice in PBS, permeabilised in PBS/0.2% Triton X-100 for 5 min and blocked in PBS/2% BSA for 30 min. Coverslips were incubated with primary followed by secondary antibodies for 1 h in PBS/2% BSA. Samples were mounted in mounting medium with DAPI (Vector Laboratories) for confocal or Prolong Antifade mounting medium (Invitrogen) for super resolution microscopy. Marker positive bacteria were enumerated by eye among at least 100 bacteria per coverslip using a wide-field microscope. Confocal images were taken with a × 63, 1.4 numerical aperture objective on either a Zeiss 710 or a Zeiss 780 microscope. Super resolution images were acquired using an Elyra S1 structured illumination microscope (Carl Zeiss Microscopy Ltd, Cambridge, UK). The system has four laser excitation sources (405nm, 488nm, 561nm and 640nm) with fluorescence emission filter sets matched to these wavelengths. SIM Images were obtained using a 63X 1.4 NA oil immersion lens with grating projections at 3 rotations and 5 phases in accordance with the manufacturer's instructions. The number of Z planes varied with sample thickness. Super resolution images were calculated from the raw data using Zeiss ZEN software.

Immunoprecipitation

For immunoprecipitation of endogenous protein with GST-fusion bait, glutathione 4b Sepharose beads were equilibrated in cold (4°C) lysis buffer (10% glycerol, 20 mM Tris HCl pH7.4, 150 mM NaCl, 0.1% Triton X-100) with protease inhibitors (1 mM PMSF, 1 mM benzamidine, 1 μg/ml aprotinin, 5 μg/ml leupeptin and 1 mM DTT). The beads were incubated end-over-end with cleared GST bacterial lysate for 2 h at 4°C and washed four times. HeLa cell lysate was obtained by lysing 3 × 10 cm tissue culture plates of confluent HeLa cells and clearing by centrifugation at 13,000 rpm in benchtop centrifuge at 4°C. Supernatant was applied to GST protein coupled beads, incubated end-over-end for 2-3 h at 4°C, washed and bound protein eluted with 100 mM glutathione in lysis buffer.

Western blotting

Cells were washed twice with ice-cold PBS and lysed in Mammalian Cell Lysis Buffer (20 mM Tris pH7.4, 150 mM NaCl, 1.0% Triton X-100, 1 mM phenylmethylsulfonyl fluoride (PMSF), 1 mM benzamidine, 2 μg/ml aprotinin and 5 μg/ml leupeptin) before clearing by centrifugation, addition of SDS loading buffer and heating to 95°C. Samples were then separated on 4%–12% denaturing gels (Thermo Fisher), transferred to PVDF membrane (Millipore) and visualized by immuno-blotting using ECL detection reagents (Amersham Bioscience).

LUMIER assay

For FLAG-based LUMIER assays relevant FLAG and Renilla Luciferase fusion expression plasmids were cotransfected into HEK293ET cells. Cells were lysed in LUMIER Lysis Buffer (20 mM Tris pH7.4, 150 mM NaCl, 0.1% Triton X-100, 5% glycerol, 1 mM phenylmethylsulfonyl fluoride (PMSF), 1 mM DTT, 1 mM benzamidine, 2 μg/ml aprotinin and 5 μg/ml leupeptin), supernatants cleared by centrifugation and FLAG-tagged proteins immobilised on anti-FLAG M2 agarose resin for 2 h at 4°C. Beads were washed four times with LUMIER Lysis Buffer (without protease inhibitors) and protein eluted with FLAG peptide. The luciferase activity in the eluate was determined using a luminometer and a Renilla Luciferase Assay Kit (Promega) and fold binding compared to FLAG-GFP control was calculated.

For GST-based LUMIER assays GST-fusion proteins were expressed in *E. coli* and mechanically lysed in lysis buffer (50 mM Tris pH 8.0, 150 mM NaCl, 1 mM EDTA, 1mM DTT, 20% glycerol, protease inhibitors (Roche)) and cleared by centrifugation. GST proteins were immobilised on glutathione 4b Sepharose beads and incubated with cell lysates derived from transiently transfected 293ET cells expressing relevant Renilla Luciferase-tagged proteins. An equivalent luciferase activity was used as the input for each IP. Samples were incubated end-over-end for 2 h at 4°C, washed four times, protein eluted with 20 mM glutathione and luciferase activity measured. Values were normalized as fold binding compared to GST only control.

Yeast Two Hybrid

Yeast two hybrid assay was carried out using NDP52 as bait using ProQuest Two-Hybrid System according to manufacturer's instructions (Life Technologies).

Reverse Transcriptase PCR

Total RNA from siRNA-treated cells was extracted using the RNeasy Plus Mini Kit (QIAGEN) followed by conversion into cDNA from a total of 300 ng RNA using the SuperScript III reverse transcriptase kit (Thermo Fisher) according to the manufacturer's protocol. Gene expression was quantified using an ULK2-specific primer pair (sense 5′- CACCTTTGAAGCCCCTGAAC and antisense 5′- CCAGTCTTTGCTCAGCTGAC)

with a Power SYBR qPCR green kit (Applied Biosystems) by following the manufacturer's protocol. Relative amounts of cDNA were calculated using the Δ Δ Ct method and normalized to β-actin cDNA levels in each sample.

Protein purification

E. coli BL21 bacteria expressing 6His-GST-FIP200ΔN1441 were mechanically lysed (20 mM Tris pH 7.4, 300 mM NaCl, 2 mM β-mercaptoethanol (βMe), 20 μg/ml DnaseI, protease inhibitors), applied to a 5 mL HisTRAP column, eluted (with a gradient of imidazole pH 8.0 from 20-400 mM) and dialysed overnight (20 mM Tris pH 7.4, 300 mM NaCl, 20 mM imidazole pH 8.0, 2 mM βMe) in the presence of TEV protease. To remove the 6xHis-TEV protease and the cleaved 6xHis-GST the sample was passed through to 5 mL Ni-NTA column. The flow through was applied to a desalting column, eluted (20 mM MES pH 6.0, 1 M NaCl, 1 mM DTT), applied to a monoS cation exchange column, the relevant fractions pooled, applied to a Superdex 75 gel filtration column (in 20 mM Tris pH 7.4, 150 mM NaCl, 1 mM DTT) and concentrated (Vivaspin, Sartorius).

Bacteria expressing 6His-GST-SINTBAD$_{aa6\text{-}85}$ were mechanically lysed (20 mM Tris pH 7.4, 150 mM NaCl, 2 mM βMe, 20 μg/ml DnaseI, protease inhibitors), applied to glutathione 4b Sepharose resin and the eluate dialysed overnight (20 mM Tris pH 7.4, 150 mM NaCl, 2 mM βme) in the presence of TEV protease. To remove the 6xHis-TEV protease and the cleaved 6xHis-GST the sample was passed through a 5ml Ni-NTA column. The flow through was diluted 1:3 in dilution buffer (20 mM Tris pH 7.4, 2 mM βMe), applied to a Resource Q anion exchange column, the relevant fractions pooled and run on a Superdex 75 gel filtration column in high salt gel filtration buffer (20 mM Tris pH 7.4, 1 M NaCl, 1 mM DTT). Protein containing fractions were pooled, applied to a desalting column and concentrated (Vivaspin, Sartorius).

Bacteria expressing 6His-GST-NDP52$_{aa20\text{-}127}$ were mechanically lysed (50 mM Tris pH 8.0, 150 mM NaCl, 10% glycerol, 2 mM DTT, 1 mM EDTA, protease inhibitors), applied to glutathione 4b Sepharose resin, washed in high salt buffer (20 mM Tris pH7.4, 300 mM NaCl, 1 mM DTT) followed by low salt buffer (20 mM Tris pH7.4, 150 mM NaCl, 1 mM DTT) and eluted (20 mM Tris pH 8.0, 150 mM NaCl, 2 mM DTT, 20 mM Glutathione). 6His-GST was cleaved from NDP52$_{aa20\text{-}127}$ with 6His-TEV protease. To remove the 6xHis-TEV protease and the cleaved 6xHis-GST the sample was applied to Ni agarose resin and the flow through containing NDP52$_{aa20\text{-}127}$ collected.

Hydrogen-Deuterium Exchange Mass Spectrometry

Protein solutions containing either 5 μM FIP200ΔN1441 or 5 μM FIP200ΔN1441 in complex with 10 μM NDP52$_{aa20\text{-}127}$ domain and 10 μM SINTBAD$_{aa6\text{-}85}$ were incubated for 1 h at 23°C in a buffer consisting of 20 mM Tris pH 7.5, 150 mM NaCl, 1 mM DTT. 10 μL of

either solution was diluted with 40 μL of D_2O Buffer (20 mM Tris pH 7.5, 150 mM NaCl, 1 mM DTT, 95.6% D_2O) for a defined period of time (3 s, 30 s) at 23°C or for 3 s on ice (a 23°C reduction in temperature relating to a ten-fold reduction in solvent exchange rate, producing a 0.3 s exchange reaction), before being quenched with 20 μL of Quench Solution (2 M guanidinium chloride, 2.4% formic acid, pH 1.6). Exchange reactions were then immediately flash frozen in liquid nitrogen and stored at −80°C prior to mass spectrometry analysis. All exchange reactions were conducted in triplicate. The final exchange reaction D_2O concentration was 76.5%.

In order to measure deuterium incorporation samples were rapidly thawed and injected onto an ultra-performance liquid chromatography (UPLC) system immersed in ice. Exchange reactions were initially digested for 3 min using an in line immobilized porcine pepsin column (Applied Biosystems; poroszyme, 2-3131-00) at 130 μL/min 0.1% formic acid, with peptides collected on a particle van-guard precolumn (Waters). Peptides were then eluted in line using an Acquity 1.7 μm particle 100 mm x 1 mm C18 UPLC Column (Waters), using a 5%–36% gradient of Buffer A (0.1% formic acid) and Buffer B (100% acetonitrile) over 20 min and injected onto a Xevo QTOF (Waters) acquiring over a mass range of 350 to 1500 *m/z* for 25 min, using an ESI source operated at a temperature of 250°C and a spray voltage of 3.0 kV.

For peptide identification three non-deuterated FIP200 protein samples were used for tandem MS/MS experiments with the UPLC system and method as described for the deuterated samples. The MS tolerance was set to 3 ppm with a MS/MS tolerance of 0.1 Da, with the retained MS/MS datasets analyzed with the Mascot search within Proteome Discoverer (Thermo Scientific). All peptides with a Mascot score > 20 were analyzed using HD-Examiner Software (Sierra Analytics). Each peptide (both deuterated and non-deuterated) was individually analyzed and manually verified for correct retention time, *m/z* range, the presence of overlapping peptide envelopes, and charge state. All percentage changes reported in the results and discussion sections are the maximal changes in HDX seen at any time-point of the analysis. No maximally deuterated control was performed due to the comparative nature of the binding experiments.

QUANTIFICATION AND STATISTICAL ANALYSIS

Data were tested for statistical significance with GraphPad Prism software. The tests performed, the sample size (n) and number of independent replicates for each experiment are indicated in the figure legends.

Molecular Cell, Volume *74*

Supplemental Information

The Cargo Receptor NDP52 Initiates Selective Autophagy by Recruiting the ULK Complex to Cytosol-Invading Bacteria

Benjamin J. Ravenhill, Keith B. Boyle, Natalia von Muhlinen, Cara J. Ellison, Glenn R. Masson, Elsje G. Otten, Agnes Foeglein, Roger Williams, and Felix Randow

Figure S1. Related to Figures 1, 2 and 6. Confirmation of siRNA mediated knockdown.

(A, B) HeLa cells transfected with the indicated siRNAs were **(A)** lysed and assayed for protein expression by Western blot using the indicated antibodies or **(B)** lysed, total mRNA extracted and the relative abundance of ULK2 and Actin mRNA assayed by RT-PCR. **(C)** HeLa cells or HEK293ET cells transiently transfected with GFP:OPTN were transfected with the indicated siRNAs, lysed and assayed for protein expression by Western blot using the indicated antibodies.

Figure S2. Related to Figure 2. GST-FIP200 fusion protein expression.

(A) Lysates were harvested from HEK293ET cells transiently transfected with plasmids encoding the indicated Luciferase-tagged proteins and assayed for protein expression by Western blot using anti Renilla Luciferase antibody. **(B)** Bacterial lysates were harvested from bacteria expressing GST:FIP200ΔN1115, GST:NDP52 or GST alone and the GST-fusion protein isolated using glutathione conjugated beads. Following washing the bound protein was eluted and visualised by gel electrophoresis and subsequent Coomassie staining.

Figure S3. Related to Figure 3. Expression of Luciferase and HIS-tagged fusion proteins

(**A-D**) HEK293ET cells transiently transfected with the indicated Luciferase tagged constructs were lysed, Luciferase activity in lysates assessed and were equalised to similar activity per unit volume. Samples were analysed by Western blot using an antibody against *Renilla* luciferase. (**E**) Bacterial lysates were prepared from either untransformed (mock) BL21 *E. coli* or expressing the indicated 6His-fusion proteins, resolved by gel electrophoresis and stained by Coomassie.

Figure S4. Related to Figure 4. Expression of Luciferase fusion proteins

(A,C) HEK293ET cells transiently transfected with the indicated Luciferase tagged constructs were lysed, Luciferase activity in lysates assessed and were equalised to similar activity per unit volume. Samples were analysed by Western blot using an antibody against *Renilla* luciferase. **(B)** Alignment of the SKICH domains of the indicated proteins. Residues conserved between all three domains are in red and those between two of the three in pink. Those residues deviant in both NDP52 and TAX1BP1 from CALCOCO1 selected for functional analysis are indicated with blue stars. **(D)** GST:FIP200ΔN1115 was assessed for its ability to bind either Luciferase:NDP52 WT or A119Q from *E. coli* in the presence or absence of $SINTBAD_{aa5-85}$, $NAP1_{aa5-75}$ or mock bacterial lysate by LUMIER assay. Error bars are the mean +/- SD of two independent experiments.

Figure S5. Related to Figure 5. Hydrogen Deuterium Exchange Mass Spectrometry

(A) The indicated FIP200, SINTBAD and NDP52 constructs were purified from bacterial lysates, resolved by gel electrophoresis and stained by Coomassie. Hatched lines indicate where lanes from a single gel were cut and pasted side-by-side. **(B)** Raw data from HDX-MS experiment comparing deuterium incorporation into FIP200ΔN1441 alone or in the presence of $SINTBAD_{aa6-85}$ and $NDP52_{aa20-127}$. Individual peptides identified are listed (S, start position; E, end position, z, charge state, #D is the maximum possible deuterium incorporation, retention time (rt), in min), % deuterium incorporation at different times shown (with the standard deviation (SD) of the triplicate repeats), the % difference in HDX calculated and the mass difference as both a percentage and mass are shown. Percentage values greater than 5%, and mass differences greater than 0.5 Da are highlighted blue. **(C)** Deuterium uptake plots of two peptides from HDX-MS binding experiment. Each peptide shows a significant reduction (p=<0.0001) in uptake on formation of the

FIP200/SINTBAD/NDP52 protein complex in comparison to FIP200 alone (Apo). Each point is the mean of three repeats, with error bars relating to standard deviation. Some error bars are smaller than the points.

Figure S6. Related to Figure 6. Complementation with NDP52 alleles and the effect of disruption of FIP200-TBK1 adaptor interactions

(A) HeLa cells stably expressing the indicated GFP-tagged proteins were infected with mCherry-*S.* Typhimurium, fixed at 1 h p.i. and confocal micrographs of representative recruitment of the GFP fusion proteins to bacteria acquired. Scale bar 10 μm. **(B, C)** HeLa cells stably expressing **(B)** FIP200ΔN1115:GFP or **(C)** GFP:SINTBAD either alone or together with the indicated siRNA-resistant alleles of NDP52 were transfected with the indicated siRNAs , lysed and assayed for protein expression by Western blot using the indicated antibodies. **(D)** HeLa cells stably expressing the indicated FIP200:GFP constructs were infected with mCherry-*S.* Typhimurium, fixed at 1 h p.i. and frequency of GFP-positive bacteria were enumerated by eye on a wide-field microscope. Mean ± SD of two independent experiments. **(E)** HeLa cells stably expressing GFP-WIPI1 were transfected with the indicated siRNAs, infected with mCherry-*S.* Typhimurium and frequency of GFP-positive bacteria were enumerated by eye on a wide-field microscope. Mean ± SD of two independent experiments.

Figure S7. Related to Figures 1-6. Initiation of selective autophagy by NDP52.

NDP52 coordinates anti-bacterial autophagy by recruiting the ULK and TBK1 kinase complexes to Galectin-8 on damaged endomembranes and by crosslinking cargo to LC3C-positive phagophore membranes. Depicted are residues, which, as described in this study, are essential for the indicated protein-protein interactions.

Supplementary Figure 1

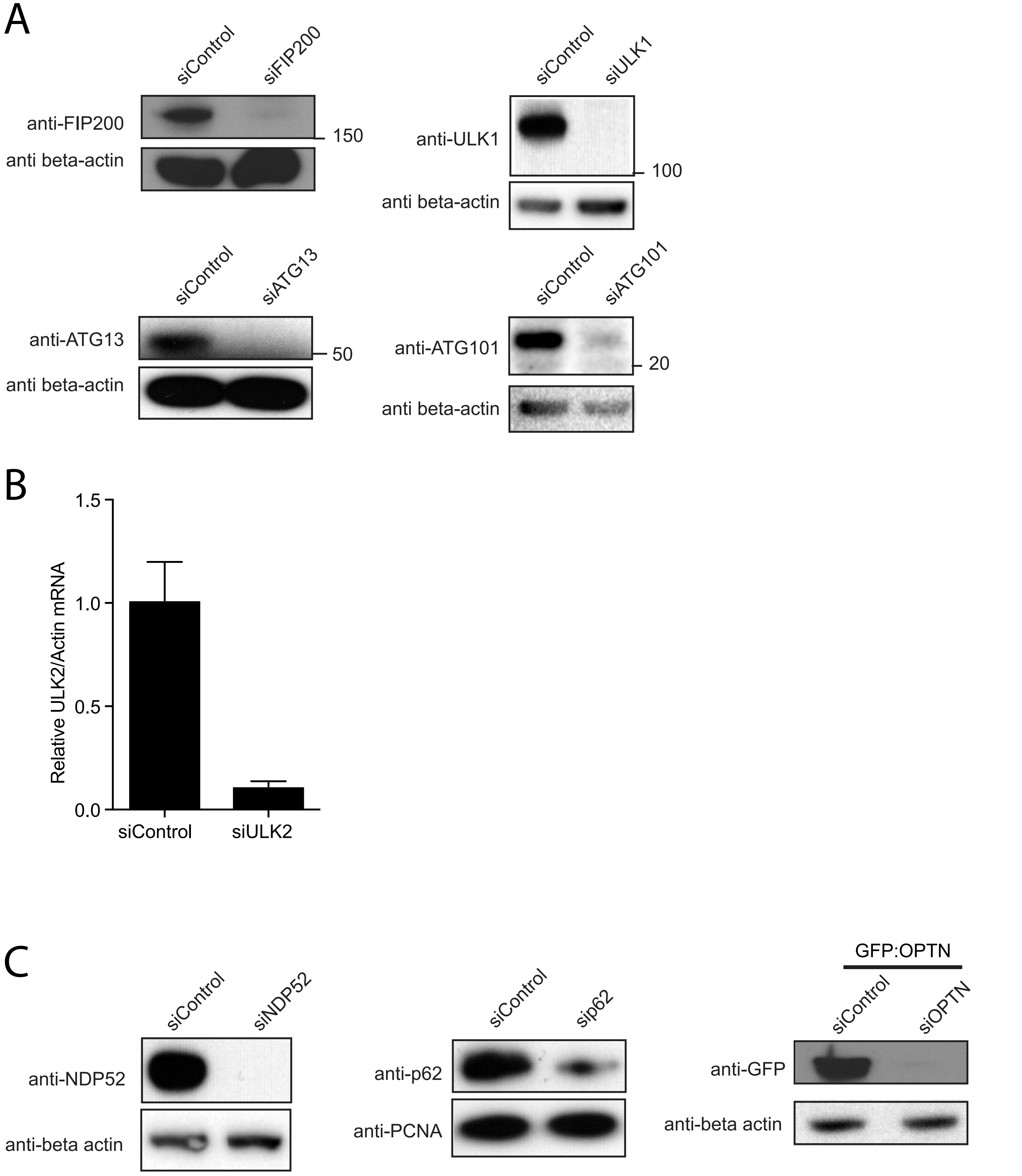

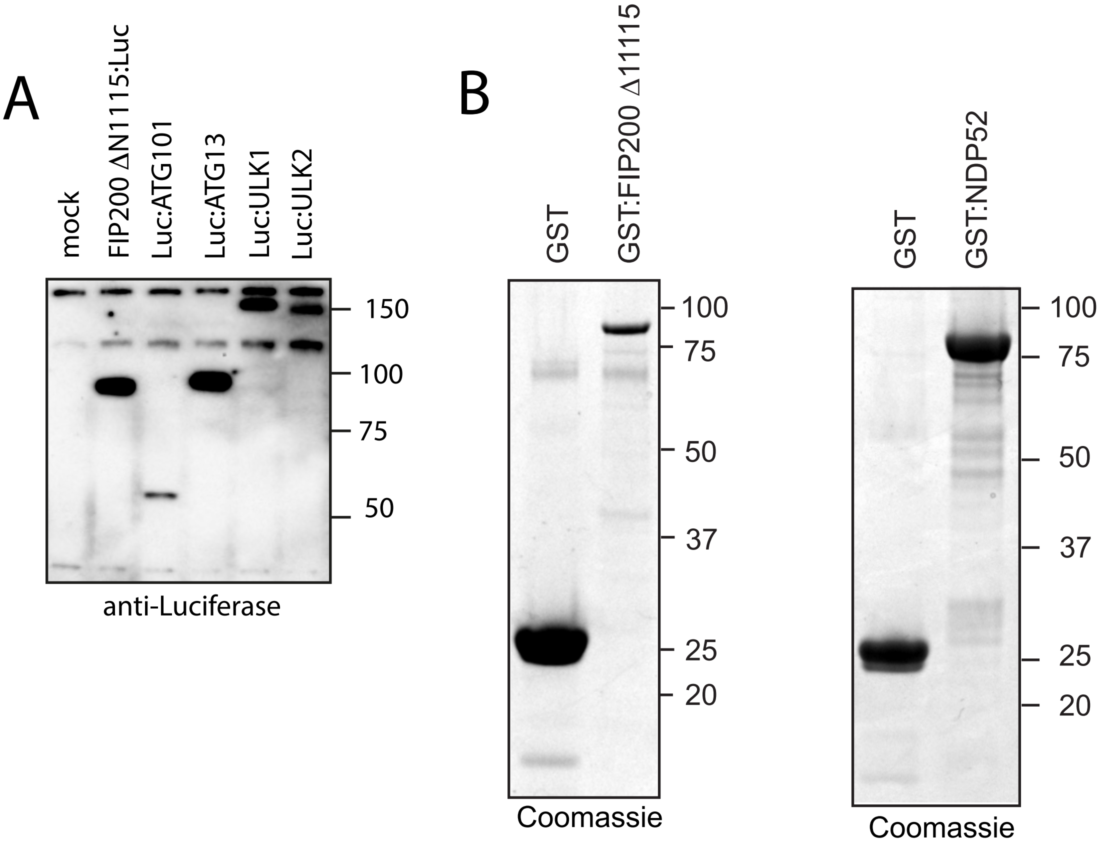
A
mock
FIP200 ΔN1115:Luc
Luc:ATG101
Luc:ATG13
Luc:ULK1
Luc:ULK2
150
100
75
50
anti-Luciferase
B
GST
GST:FIP200 Δ11115
100
75
50
37
25
20
Coomassie
GST
GST:NDP52
100
75
50
37
25
20
Coomassie

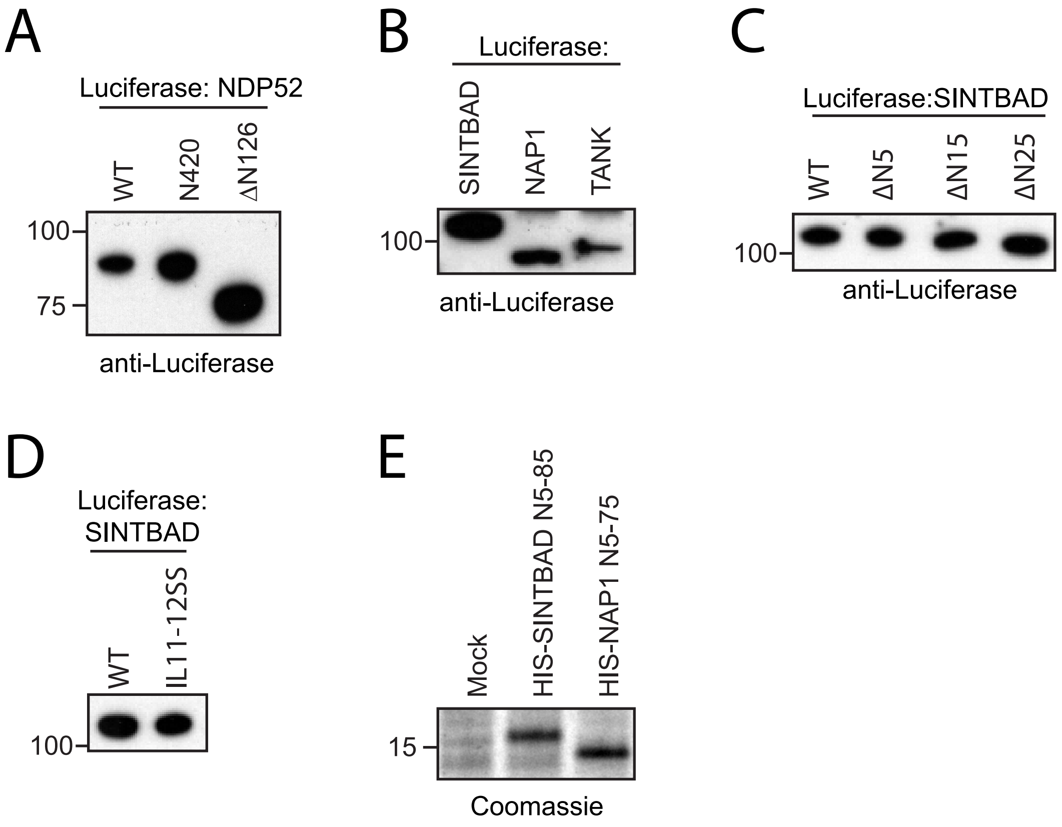
A
Luciferase: NDP52
WT
N420
ΔN126
100
75
anti-Luciferase
B
Luciferase:
SINTBAD
NAP1
TANK
100
anti-Luciferase
C
Luciferase:SINTBAD
WT
ΔN5
ΔN15
ΔN25
100
anti-Luciferase
D
Luciferase:
SINTBAD
WT
IL11-12SS
100
E
Mock
HIS-SINTBAD N5-85
HIS-NAP1 N5-75
15
Coomassie

A

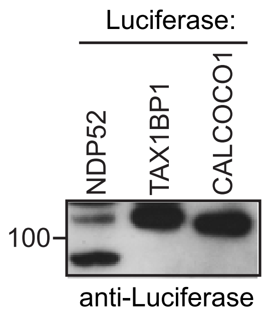
Luciferase:
NDP52
TAX1BP1
CALCOCO1
100
anti-Luciferase

B

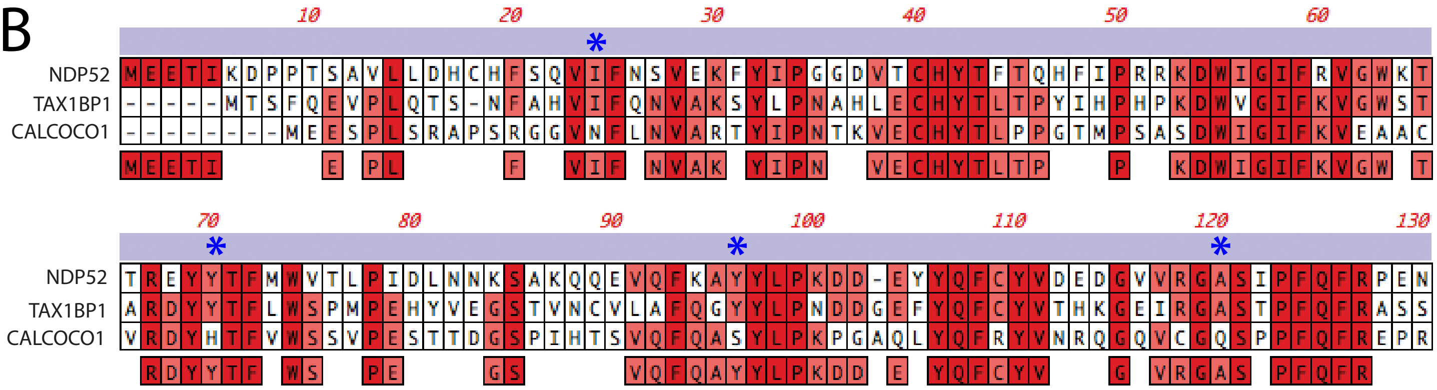
10
20
30
40
50
60
NDP52 M E E T I K D P P T S A V L L D H C H F S Q V I F N S V E K F Y I P G G D V T C H Y T F T Q H F I P R R K D W I G I F R V G W K T
TAX1BP1 - - - - - - M T S F Q E V P L Q T S - N F A H V I F Q N V A K S Y L P N A H L E C H Y T L T P Y I H P H P K D W V G I F K V G W S T
CALCOCO1 - - - - - - - - - M E E S P L S R A P S R G G V N F L N V A R T Y I P N T K V E C H Y T L P P G T M P S A S D W I G I F K V E A A C
M E E T I E P L F V I F N V A K Y I P N V E C H Y T L T P P K D W I G I F K V G W T
70
80
90
100
110
120
130
NDP52 T R E Y Y T F M W V T L P I D L N N K S A K Q Q E V Q F K A Y Y L P K D D - E Y Y Q F C Y V D E D G V V R G A S I P F Q F R P E N
TAX1BP1 A R D Y Y T F L W S P M P E H Y V E G S T V N C V L A F Q G Y Y L P N D D G E F Y Q F C Y V T H K G E I R G A S T P F Q F R A S S
CALCOCO1 V R D Y H T F V W S S V P E S T T D G S P I H T S V Q F Q A S Y L P K P G A Q L Y Q F R Y V N R Q G Q V C G Q S P P F Q F R E P R
R D Y Y T F W S P E G S V Q F Q A Y Y L P K D D E Y Q F C Y V G V R G A S P F Q F R

C

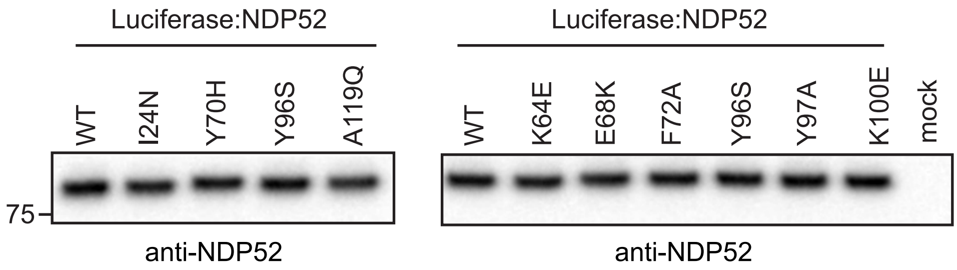
Luciferase:NDP52
WT
I24N
Y70H
Y96S
A119Q
75
anti-NDP52
Luciferase:NDP52
WT
K64E
E68K
F72A
Y96S
Y97A
K100E
mock
anti-NDP52

D

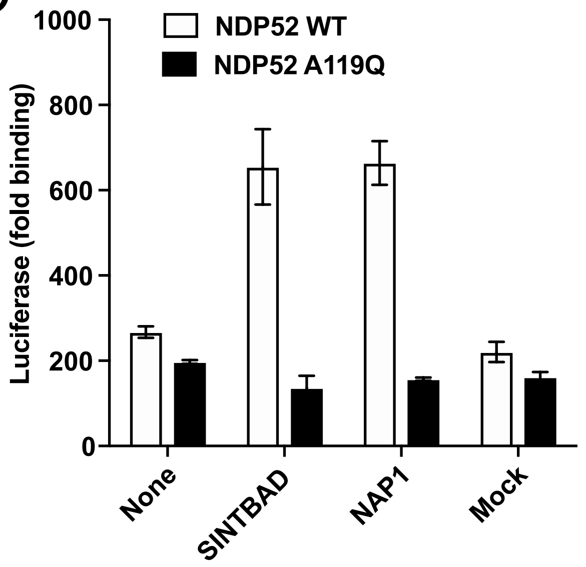
NDP52 WT
NDP52 A119Q
Luciferase (fold binding)
1000
800
600
400
200
0
None
SINTBAD
NAP1
Mock

Supplementary Figure 5

A

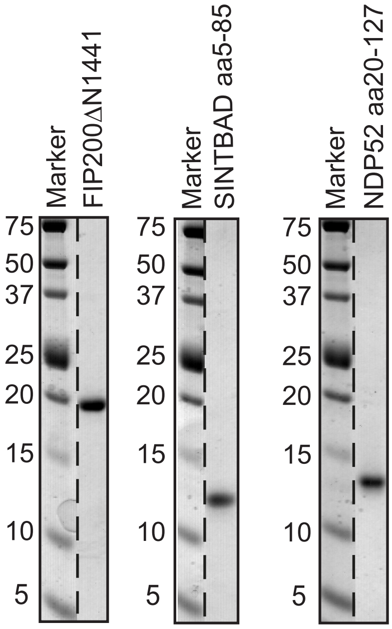

B

Peptide					FIP200						FIP200+SINTBAD +NDP52						% difference in HDX						Da difference in HDX						Sequence
S	E	z	#D	rt (min)	0.3 s	SD	3 s	SD	30 s	SD	0.3 s	SD	3 s	SD	30 s	SD	0.3 s	SD	3 s	SD	30 s	SD	0.3 s	SD	3 s	SD	30 s	SD	
1443	1451	2	7	9.1	81	2	82	1	80	1	81	1	83	2	83	0	0	3	-1	2	-3	1	0.0	0.2	-0.1	0.2	-0.2	0.1	MSVQENIHM
1448	1461	3	12	9.5	31	1	46	1	56	1	29	1	46	2	57	1	2	2	0	3	-1	2	0.3	0.2	0.0	0.4	-0.1	0.3	NIHMLSEEKQRIML
1452	1461	2	8	8.8	28	1	51	1	69	1	25	2	49	1	67	1	4	2	1	2	2	2	0.3	0.2	0.1	0.2	0.1	0.2	LSEEKQRIML
1452	1462	2	9	10.4	23	0	45	1	67	0	21	2	45	1	68	1	2	2	0	2	-1	1	0.2	0.2	0.0	0.2	-0.1	0.1	LSEEKQRIMLL
1453	1461	2	7	8.8	30	1	55	1	75	0	26	2	53	1	72	2	4	2	2	2	2	2	0.3	0.2	0.1	0.1	0.2	0.1	SEEKQRIML
1453	1462	2	8	8.8	28	1	51	1	69	1	25	2	49	1	67	1	4	2	1	2	2	2	0.3	0.2	0.1	0.2	0.1	0.2	SEEKQRIMLL
1454	1461	2	6	8.7	35	1	58	0	76	1	31	2	56	1	77	1	4	3	2	1	0	1	0.2	0.2	0.1	0.1	0.0	0.1	EEKQRIML
1462	1468	1	5	9.7	6	1	36	2	77	1	5	0	30	0	73	1	1	1	6	2	4	3	0.1	0.0	0.3	0.1	0.2	0.1	LERTLQL
1462	1468	2	5	9.7	6	0	36	2	77	2	5	0	31	1	71	2	1	0	5	3	6	3	0.1	0.0	0.2	0.2	0.3	0.2	LERTLQL
1462	1480	3	17	7.2	8	1	41	2	67	1	6	0	35	2	64	1	2	1	6	3	3	2	0.4	0.2	1.0	0.6	0.6	0.3	LERTLQLKEEENKRLNQRL
1462	1480	4	17	7.3	8	0	41	2	67	1	6	1	36	0	64	2	2	1	5	3	3	3	0.4	0.2	0.8	0.4	0.5	0.4	LERTLQLKEEENKRLNQRL
1463	1468	1	4	9.0	6	0	35	2	77	0	5	1	31	1	72	2	1	1	4	3	5	2	0.1	0.0	0.2	0.1	0.2	0.1	ERTLQL
1463	1480	4	16	7.0	9	0	42	2	68	0	6	1	36	1	65	2	2	1	5	2	2	2	0.4	0.1	0.8	0.3	0.4	0.3	ERTLQLKEEENKRLNQRL
1463	1480	5	16	7.1	10	0	41	1	67	1	7	0	36	1	64	2	2	1	4	2	3	2	0.4	0.1	0.7	0.3	0.5	0.4	ERTLQLKEEENKRLNQRL
1468	1479	3	10	4.9	13	1	48	1	64	0	10	1	44	1	63	0	4	2	4	2	1	1	0.4	0.2	0.4	0.2	0.1	0.1	LKEEENKRLNQR
1468	1480	4	11	5.2	12	2	46	2	62	1	9	1	40	1	61	1	4	3	6	3	2	2	0.4	0.3	0.6	0.3	0.2	0.2	LKEEENKRLNQRL
1469	1480	2	10	4.8	13	1	47	2	63	0	10	1	43	1	62	1	4	2	4	2	1	1	0.4	0.2	0.4	0.2	0.1	0.1	KEEENKRLNQRL
1481	1500	3	18	6.1	30	1	36	2	34	1	30	1	36	3	36	1	1	2	-1	5	-2	2	0.1	0.3	-0.1	0.8	-0.3	0.4	MSQSMSSVSSRHSEKIAIRD
1481	1508	4	26	11.4	13	0	17	1	20	1	11	0	17	1	18	0	3	1	1	2	2	2	0.7	0.2	0.1	0.5	0.5	0.5	MSQSMSSVSSRHSEKIAIRDFQVGDLVL
1486	1506	3	19	9.7	13	3	20	0	25	1	12	0	20	1	24	0	1	4	0	1	1	2	0.2	0.7	0.0	0.2	0.2	0.4	SSVSSRHSEKIAIRDFQVGDL
1488	1500	3	11	4.7	22	1	28	1	28	1	20	0	28	2	28	1	2	2	1	3	0	1	0.2	0.2	0.1	0.3	0.0	0.2	VSSRHSEKIAIRD
1489	1500	3	10	4.6	23	1	29	1	29	1	18	1	28	1	28	1	4	2	1	1	1	2	0.4	0.2	0.1	0.1	0.1	0.2	SSRHSEKIAIRD
1506	1510	1	3	13.4	1	0	5	0	18	0	1	0	5	0	17	0	0	0	0	0	0	1	0.0	0.0	0.0	0.0	0.0	0.0	LVLII
1509	1520	2	10	10.2	11	0	17	0	22	0	10	0	15	1	21	1	2	1	2	1	1	1	0.2	0.1	0.2	0.1	0.1	0.1	IILDERHDNYVL
1512	1520	2	7	9.0	12	1	19	0	23	0	10	0	17	1	23	0	2	1	2	1	0	1	0.1	0.1	0.2	0.1	0.0	0.0	DERHDNYVL
1512	1521	2	8	11.4	10	0	14	2	16	1	7	1	12	0	15	1	2	1	2	2	1	1	0.2	0.1	0.1	0.2	0.1	0.1	DERHDNYVLF
1521	1527	1	4	10.3	35	2	71	2	88	1	26	3	66	1	86	1	9	5	5	3	2	2	0.4	0.2	0.2	0.1	0.1	0.1	FTVSPTL
1522	1528	1	4	9.3	31	2	59	2	70	1	24	2	58	1	69	1	7	4	1	3	0	2	0.3	0.2	0.0	0.1	0.0	0.1	TVSPTLY
1528	1535	2	6	10.3	7	1	19	0	36	1	4	1	13	1	30	2	3	1	6	1	5	2	0.2	0.1	0.4	0.1	0.3	0.1	YFLHSESL
1528	1538	2	8	11.8	11	1	22	1	40	0	9	0	15	1	31	1	2	1	7	1	8	1	0.2	0.1	0.6	0.1	0.7	0.1	YFLHSESLPAL
1539	1561	3	19	9.6	32	1	33	1	36	1	29	1	32	3	33	1	3	2	2	3	2	2	0.6	0.3	0.3	0.6	0.5	0.4	DLKPGEGASGASRRPWVLGKVME
1541	1556	3	13	10.2	46	1	49	0	55	1	43	1	47	2	51	1	3	2	2	3	4	2	0.4	0.2	0.3	0.3	0.5	0.2	KPGEGASGASRRPWVL
1547	1556	2	7	10.1	57	1	63	1	72	1	53	1	60	3	69	2	4	2	3	3	3	3	0.3	0.2	0.2	0.2	0.2	0.2	SGASRRPWVL
1547	1563	4	14	9.0	29	2	31	1	37	1	27	2	29	2	32	2	2	3	2	3	5	3	0.3	0.5	0.3	0.5	0.7	0.4	SGASRRPWVLGKVMEKE
1557	1563	1	5	4.0	17	0	19	2	18	0	19	1	19	1	20	1	-2	2	0	3	-2	1	-0.1	0.1	0.0	0.2	-0.1	0.1	GKVMEKE
1557	1582	4	23	8.3	45	1	52	1	55	0	32	1	41	1	47	1	13	1	11	2	8	1	3.0	0.3	2.5	0.4	1.8	0.2	GKVMEKEYCQAKKAQNRFKVPLGTKF
1562	1582	4	18	7.6	47	1	53	1	56	0	32	1	40	1	46	0	15	2	12	2	10	1	2.8	0.3	2.2	0.4	1.8	0.1	KEYCQAKKAQNRFKVPLGTKF
1568	1582	4	12	7.7	42	0	51	0	55	0	28	1	37	1	43	1	14	1	14	1	12	1	1.7	0.1	1.7	0.2	1.5	0.2	KKAQNRFKVPLGTKF
1583	1590	2	6	9.2	18	1	30	0	33	0	14	1	28	0	32	1	4	2	1	1	2	1	0.2	0.1	0.1	0.0	0.1	0.1	YRVKAVSW
1583	1593	3	9	6.7	36	1	45	0	46	0	32	2	42	2	44	2	4	3	2	2	2	2	0.4	0.2	0.2	0.2	0.2	0.2	YRVKAVSWNKK
1583	1594	3	10	6.8	35	1	43	1	44	0	31	1	42	2	42	2	4	2	2	3	2	2	0.4	0.2	0.2	0.3	0.2	0.2	YRVKAVSWNKKV

C

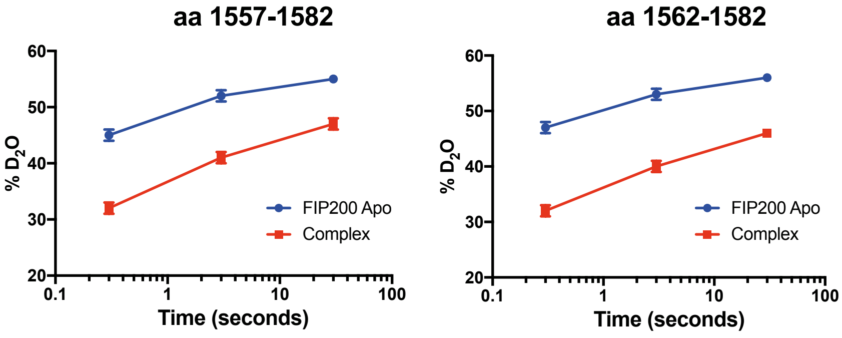

Supplementary Figure 6

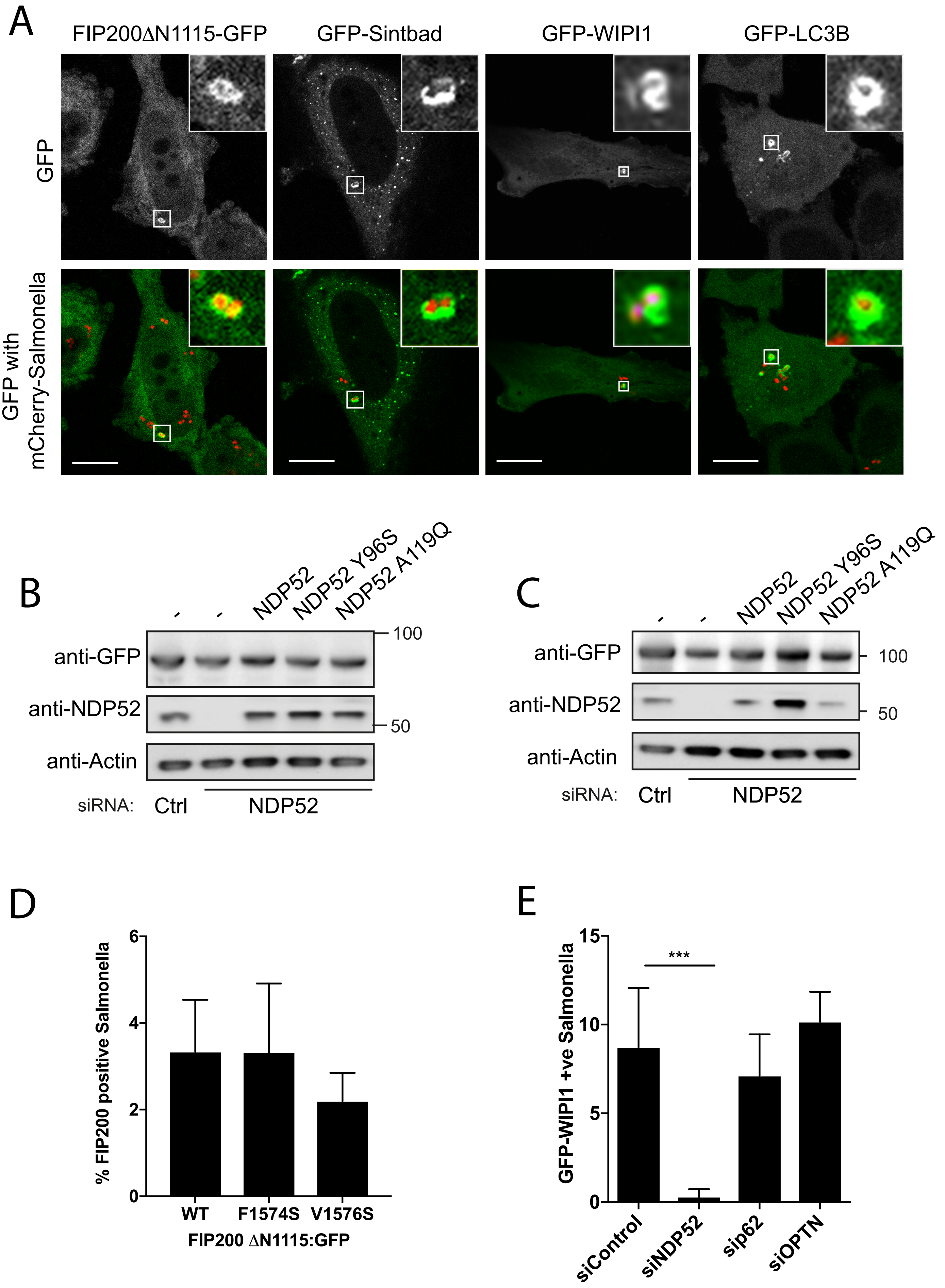

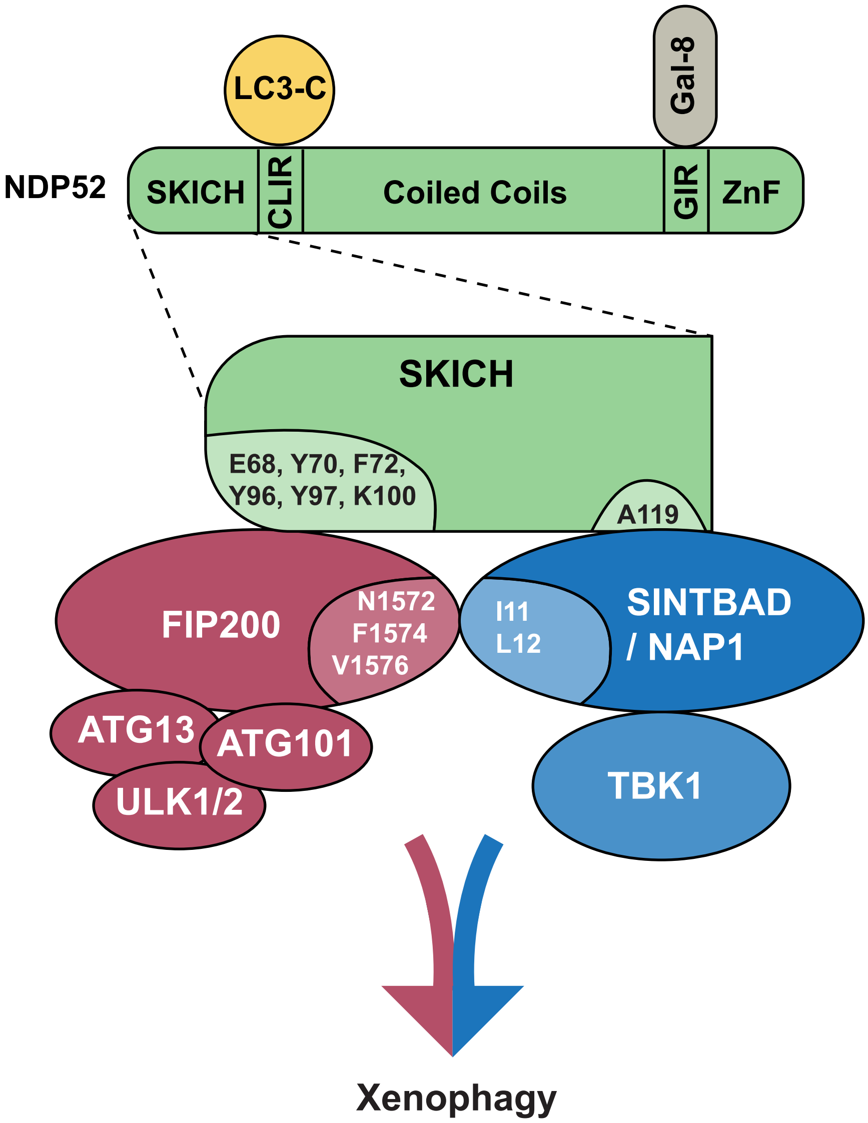
LC3-C
Gal-8
NDP52
SKICH
CLIR
Coiled Coils
GIR
ZnF
SKICH
E68, Y70, F72,
Y96, Y97, K100
A119
FIP200
N1572
F1574
V1576
I11
L12
SINTBAD
/ NAP1
ATG13
ATG101
ULK1/2
TBK1
Xenophagy
